# Association between serum carotenoid concentrations and risk of major age-related eye diseases among middle-aged and older adults

**DOI:** 10.3389/fmed.2025.1596799

**Published:** 2025-12-01

**Authors:** Songtian Che, Yan Ma, Jinfeng Cao

**Affiliations:** 1 Department of Ophthalmology, The Second Hospital of Jilin University, Changchun, China; 2 Jilin Provincial Engineering Laboratory of Ophthalmology, Changchun, China

**Keywords:** serum carotenoids, cataract, glaucoma, diabetic retinopathy, age-related macular degeneration, NHANES

## Abstract

**Background:**

Age-related eye diseases are the main causes of progressive and irreversible vision loss in aging populations worldwide. Carotenoids, as a group of common natural antioxidants, can suppress free radicals produced by complex physiological reactions, thereby protecting the eyes from the effects of oxidative stress, cell apoptosis, and mitochondrial dysfunction. The present study aims to explore the association between serum carotenoid concentrations and risk of major age-related eye diseases among middle-aged and older adults in the United States.

**Methods:**

This study involved 1,478 participants aged ≥50 years from the 2005–2006 cycles of the National Health and Nutrition Examination Survey (NHANES). Multivariate logistic regression was used to estimate the odds ratios (ORs) and 95% confidence intervals (CIs) of prevalence of cataract, glaucoma, diabetic retinopathy, and age-related macular degeneration (AMD) in relation to serum carotenoid concentrations.

**Results:**

Compared to participants in the first quartile, those in highest quartile of serum *α*-carotene (OR: 0.37; 95% CI: 0.21–0.64), *β*-carotene (OR: 0.57; 95% CI: 0.33–0.95), lutein/zeaxanthin (OR: 0.45; 95% CI: 0.27–0.76), and total carotenoid (OR: 0.58; 95% CI: 0.35–0.97) were negatively associated with risk of cataract; those in highest quartile of serum *β*-carotene (OR: 0.30; 95% CI: 0.11–0.77) and β-cryptoxanthin (OR: 0.28; 95% CI: 0.12–0.68) were negatively associated with risk of diabetic retinopathy; and those in highest quartile of lycopene (OR: 0.37; 95% CI: 0.18–0.78) was negatively associated with risk of AMD. In addition, subgroup analysis results indicated that participants in highest quartile of serum *α*-carotene (OR: 0.16; 95% CI: 0.08–0.32), *β*-carotene (OR: 0.40; 95% CI: 0.21–0.75), lycopene (OR: 0.46; 95% CI: 0.24–0.87), lutein/zeaxanthin (OR: 0.45; 95% CI: 0.25–0.84), and total carotenoid (OR: 0.41; 95% CI: 0.22–0.77) concentrations were negatively associated with risk of any ocular disease among female participants. By contrast, no associations were observed among male participants.

**Conclusion:**

Our study demonstrated that higher serum concentrations of carotenoids were negatively associated with the risk of age-related eye diseases, particularly among middle-aged and older female participants.

## 1 Introduction

With the rapid growth of middle-aged and older adult populations, the prevalence of common age-related eye diseases has increased, including cataract, glaucoma, diabetic retinopathy, and age-related macular degeneration (AMD), which are the main causes of progressive and irreversible vision loss in aging populations worldwide (1–3). Globally, 285 million people have moderate to severe visual impairment or blindness, of whom 65% of the visually impaired and 82% of all blind people are aged 50 or older (4). In the United States, the prevalence of most eye diseases increases with age among individuals aged ≥50 years, and such diseases impose a substantial socioeconomic burden (5). Furthermore, age-related eye diseases can reduce the quality of life of older adults and independently increase their mortality (6–8). Because of their overall health implications, age-related eye diseases are a major health concern among middle-aged and older adults, and new strategies for preventing or delaying disease progression must be identified.

Studies have reported that the cells of the tissues of the eyes are sensitive to oxidative stress, triggering and exacerbating age-related ocular abnormalities (9). Carotenoids are a group of common natural antioxidants and fat-soluble phytochemicals that are synthesized only by specific microorganisms and plants (10, 11). To the best of our knowledge, *α*-carotene, *β*-carotene, β-cryptoxanthin, lycopene, lutein, and zeaxanthin account for more than 95% of the carotenoids in circulation (11, 12). Because ocular carotenoids absorb light in the visible light region, they protect the retina and lens from the potential photochemical damage caused by light exposure (13, 14). Carotenoids can also suppress the free radicals produced by complex physiological reactions, thereby protecting the eyes from the effects of oxidative stress, cell apoptosis, mitochondrial dysfunction, and inflammation (13). Several previous studies have reported that dietary carotenoid supplementation had a potential beneficial association with reduced risk of cataract, preserved macular health in glaucoma and promoted retinal health, and improved visual function in diabetic retinopathy (15–18). In addition, the Age-Related Eye Diseases Study (AREDS) Research Group demonstrated that dietary lutein/zeaxanthin supplementation was significantly associated with late AMD progression and can serve as an appropriate replacement for beta carotene, which increased the risk of lung cancer (19, 20).

To date, the majority of previous studies have focused on the association between dietary carotenoid intake and eye diseases (21, 22), whereas epidemiological evidence regarding the relationship between serum carotenoid concentrations and the risk of age-related eye diseases remains limited. Serum concentrations provide a direct measure of absorbed, metabolically processed carotenoids, reflecting interindividual differences in digestion, absorption, and metabolism that dietary assessments cannot capture. A cross-sectional study demonstrated that a higher concentration of serum *α*-carotene is associated with a lower risk of diabetic retinopathy (23). Furthermore, higher blood concentrations of *α*-carotene, *β*-carotene, β-cryptoxanthin, lycopene, and lutein/zeaxanthin are associated with a lower risk of cataract among individuals aged ≥50 years (24). A case–control study reported that higher serum concentrations of carotenoids, particularly zeaxanthin and lycopene, were associated with a lower likelihood of developing exudative AMD in an older Chinese population (25). Notably, most of the aforementioned studies have mainly focused on the association between the serum carotenoid concentration and single eye diseases in Asian populations. To date, few studies have comprehensively explored the association between serum carotenoids and various age-related eye diseases among populations in the United States.

In the present population-based cross-sectional study, we examined the association of the serum carotenoid concentration with the risk of major age-related eye diseases, including cataract, glaucoma, diabetic retinopathy, and AMD, in a nationally representative sample of the middle-aged and older adult population in the United States.

## 2 Materials and methods

### 2.1 Study population

The National Health and Nutrition Examination Survey (NHANES) is an ongoing cross-sectional study conducted by the National Center for Health Statistics (NCHS) at the Centers for Disease Control and Prevention (CDC) to assess the health and nutritional status of a nationally representative sample of the civilian population in the United States. Data from the 2005–2006 cycle of the NHANES were used for this analysis. The protocols for the NHANES study were approved by the Research Ethics Review Board of the NCHS. Each participant provided written informed consent.

In the NHANES 2005–2006, there were a total of 10,348 participants, and the present analysis was limited to 2,214 participants aged 50 and older. We excluded participants with missing data for serum carotenoid concentrations (*n* = 220) and major eye diseases (*n* = 516), including cataract, glaucoma, diabetic retinopathy, and AMD. Finally, 1,478 participants were included in the present study.

### 2.2 Assessment of carotenoids

Serum carotenoids, including *α*-carotene, *β*-carotene, β-cryptoxanthin, lycopene, and lutein/zeaxanthin, were quantified at the CDC’s National Center for Environmental Health using validated high-performance liquid chromatography (HPLC) with multiwavelength photodiode-array absorbance detection. The laboratory procedures involved mixing serum with a buffer and ethanol containing internal standards—retinyl butyrate and nonapreno-beta-carotene (C45)—followed by extraction into hexane. The combined hexane extracts were then redissolved in ethyl acetate, diluted in mobile phase, and injected onto a C18 reversed-phase column for isocratic elution. Carotenoids were detected by absorbance at 450 nm. The coefficient of variation (CV) was generally below 5% for *β*-carotene and below 20% for minor carotenoids. Total serum carotenoid concentration was calculated as the sum of the five individual carotenoids. Detailed quality control methods and analytical protocols have been described elsewhere (12, 26).

### 2.3 Assessment of major eye disease

The NHANES database provided retinal imaging results, which were used to examine the presence of diabetic retinopathy and AMD. Diabetic retinopathy was determined by any signs of retinopathy and the diagnosis of diabetes (27). Based on the fundus photographs, the retinopathy was graded by four levels, including no retinopathy, mild non-proliferative retinopathy (NPR), moderate, or severe NPR. Diabetes was defined as a self-report of a previous diagnosis of the disease or a hemoglobin A_1c_ (HbA_1c_) level≥6.5% according to the American Diabetes Association’s diagnostic criteria for diabetes (28). According to the modified Wisconsin Age-Related Maculopathy Grading Classification Scheme, early AMD is defined by the presence or absence of drusen and/or pigmentary abnormalities; late AMD is defined by exudative AMD signs and/or geographic atrophy. In the present study, participants were divided into two groups: no AMD and AMD. A range of quality control measures was implemented to ensure the accuracy and reliability of retinal image grading. Fundus images were assessed by a team of nine trained graders, including a preliminary grading coordinator, two preliminary graders, and six detail graders. Grading was conducted in a semi-quantitative manner using EyeQ Lite software, with high-resolution monitors for detailed evaluation. The process involved three stages: an initial review for detectable pathology, preliminary grading, and detailed grading. Each image was independently evaluated by at least two graders. In case of disagreement, the image was evaluated by an adjudicator who made a final decision. Further details are available in the NHANES Digital Grading Protocol.

Furthermore, NHANES provided self-reported personal interview data on vision status, including cataract and glaucoma. The cataract status in all participants was based on their answers to the question, “Have you ever had a cataract operation?” Glaucoma was defined by the question: “Have you ever been told you had glaucoma, sometimes called high pressure in eyes?” The questions were asked using the Computer-Assisted Personal Interviewing system (CAPI), which was programmed with built-in consistency checks to reduce data entry errors.

### 2.4 Statistical analysis

Data were expressed as mean ± standard deviation (SD) of continuous variables and numbers (percentages) of categorical variables. Demographic characteristics between quartiles of serum total carotenoid concentrations were compared by one-way ANOVA tests for continuous variables and chi-square tests for categoric variables. Univariate and multivariate logistic regression analyses were used to estimate the odds ratios (ORs) and 95% confidence intervals (CIs) of prevalence of cataract, glaucoma, diabetic retinopathy, and AMD in relation to serum carotenoid concentrations. *p*-values for trend were obtained by including the quartile number as a continuous variable in the regression model. The multivariate adjusted model was adjusted for age (continuous), sex (male or female), race/ethnicity (non-Hispanic white, black, Mexican-American, other Hispanic, or other race/ethnicity), education level (less than high school, high school or equivalent, college or above, or missing), family income-to-poverty ratio (FIR) (<1.3, 1.3 to ≤3.5, >3.5, or missing), body mass index (BMI) (<18.5, 18.5 to <25, 25 to <30, ≥30 kg/m^2^, or missing), drinking status (non-drinker or drinker), smoking status (never smoker, former smoker, current smoker, or missing), HbA_1c_ (<6.5%, ≥6.5%, or missing), hypertension (yes, no, or missing), and hypercholesterolemia (yes, no, or missing). We selected these confounders on the basis of their associations with the outcomes of interest or a change in the effect estimate of more than 10% (29, 30). Model goodness-of-fit was assessed using Akaike Information Criterion (AIC) and residual plots. We used the restricted cubic spline regression models to investigate dose–response associations between serum *α*-carotene, *β*-carotene, β-cryptoxanthin, lycopene, lutein/zeaxanthin, and total carotenoid concentrations and risk of any ocular disease. Sensitivity analyses by excluding participants with missing data on covariates were also conducted. Furthermore, interaction and stratified analyses were conducted according to sex (male vs. female) and smoking status (never smoker vs. former or current smoker), and *P* for interaction was calculated using the likelihood ratio test. Significance for pre-specified subgroup interactions (sex and smoking status) was assessed using a Bonferroni-corrected threshold of a *p*-value of < 0.025 to account for multiple testing. All statistical analyses were conducted using R Statistical Software (Version 4.2.2).

## 3 Results

### 3.1 Demographic characteristics of the study participants

The demographic characteristics of participants are summarized in Table 1. Among 1,478 participants, there were 775 (52.44%) males and 703 (47.56%) females, with an average age of 65.34 ± 10.13 years. There were differences in age, sex, race, education, FIR, BMI, smoking status, and proportion of alcohol drinkers, diabetes, and hypertension between quartiles of serum total carotenoid concentrations (all *p* < 0.05). Participants with higher serum total carotenoid concentrations were younger, more likely to be female and alcohol drinkers, less likely to be non-Hispanic whites, obese, and current smokers, and tended to have greater education and FIR level, a lower proportion of HbA1 ≥ 6.5% and hypertension. Comparison of serum carotenoid concentrations among participants, stratified by sex, is shown in Supplementary Table 1. The results showed that female participants have lower carotenoid levels compared to male participants.

**Table 1:** Characteristics of participants by quartiles of serum total carotenoid concentrations.

	Serum total carotenoid concentrations (μmol/L)				
	Total (*n* = 1,478)	Quartile 1 (*n* = 370)	Quartile 2 (*n* = 368)	Quartile 3 (*n* = 370)	Quartile 4 (*n* = 370)
Socioeconomics and health factors					
Age, year	65.34 ± 10.13	66.52 ± 10.45	65.22 ± 9.85	65.35 ± 10.20	64.28 ± 9.94
Male sex, *n* (%)	775 (52.44)	219 (59.19)	193 (52.45)	198 (53.51)	165 (44.59)
Race/Ethnicity, *n* (%)					
Non-Hispanic white	892 (60.35)	240 (64.86)	217 (58.97)	215 (58.11)	220 (59.46)
Black	305 (20.64)	78 (21.08)	89 (24.18)	70 (18.92)	68 (18.38)
Mexican American	217 (14.68)	39 (10.54)	53 (14.40)	68 (18.38)	57 (15.41)
Other Hispanic	27 (1.83)	7 (1.89)	2 (0.54)	8 (2.16)	10 (2.70)
Other race/ethnicity	37 (2.50)	6 (1.62)	7 (1.90)	9 (2.43)	15 (4.05)
Education, *n* (%)					
Less than high school	218 (14.75)	66 (17.84)	56 (15.22)	48 (12.97)	48 (12.97)
High school or equivalent	600 (40.60)	174 (47.03)	169 (45.92)	147 (39.73)	110 (29.73)
College or above	660 (44.65)	130 (35.14)	143 (38.86)	175 (47.30)	212 (57.30)
Family income-to-poverty ratio, *n* (%)					
<1.3	319 (22.80)	118 (33.43)	78 (22.41)	69 (19.77)	54 (15.47)
1.3 to ≤3.5	564 (40.31)	160 (45.33)	137 (39.37)	141 (40.40)	126 (36.10)
>3.5	516 (36.88)	75 (21.25)	133 (38.22)	139 (39.83)	169 (48.42)
Missing	79 (5.35)	17 (4.59)	20 (5.43)	21 (5.68)	21 (5.68)
Body mass index, *n* (%)					
<18.5 kg/m^2^	21 (1.43)	7 (1.93)	2 (0.55)	3 (0.82)	9 (2.45)
18.5 to <25 kg/m^2^	374 (25.55)	81 (22.38)	73 (19.95)	79 (21.47)	141 (38.32)
25 to <30 kg/m^2^	539 (36.82)	122 (33.70)	134 (36.61)	149 (40.49)	134 (36.41)
≥30 kg/m^2^	530 (36.20)	152 (41.99)	157 (42.90)	137 (37.23)	84 (22.83)
Missing	14 (0.95)	8 (2.16)	2 (0.54)	2 (0.54)	2 (0.54)
Smoking status, *n* (%)					
Never smoker	660 (44.81)	125 (33.88)	165 (45.21)	171 (46.22)	199 (53.93)
Former smoker	548 (37.20)	146 (39.57)	121 (33.15)	148 (40.00)	133 (36.04)
Current smoker	265 (17.99)	98 (26.56)	79 (21.64)	51 (13.78)	37 (10.03)
Missing	5 (0.34)	1 (0.27)	3 (0.82)	0 (0.00)	1 (0.27)
Alcohol drinker, *n* (%)	973 (67.29)	245 (67.68)	221 (61.39)	243 (67.13)	264 (72.93)
Missing	32 (2.17)	8 (2.16)	8 (2.17)	8 (2.16)	8 (2.16)
Hemoglobin A_1c_ ≥ 6.5%, *n* (%)	245 (16.58)	87 (23.51)	70 (19.02)	47 (12.70)	41 (11.08)
Missing	3 (0.20)	1 (0.27)	1 (0.27)	0 (0.00)	1 (0.27)
Hypertension, *n* (%)	745 (50.54)	209 (56.49)	203 (55.62)	168 (45.53)	165 (44.59)
Missing	4 (0.27)	0 (0.00)	3 (0.82)	1 (0.27)	0 (0.00)
Hypercholesterolemia, *n* (%)	677 (53.65)	153 (49.35)	174 (55.59)	177 (56.37)	173 (53.23)
Missing	216 (14.61)	60 (16.22)	55 (14.95)	56 (15.14)	45 (12.16)
Cancer, *n* (%)	222 (15.02)	59 (15.95)	49 (13.32)	56 (15.14)	58 (15.68)
Missing	3 (0.20)	2 (0.54)	0 (0.00)	1 (0.27)	0 (0.00)
Major eye diseases					
Cataract	230 (15.56)	76 (20.54)	63 (17.12)	48 (12.97)	43 (11.62)
Glaucoma	113 (7.65)	35 (9.46)	31 (8.42)	22 (5.95)	25 (6.76)
Diabetic retinopathy	94 (6.36)	33 (8.92)	29 (7.88)	19 (5.14)	13 (3.51)
Age-related macular degeneration	110 (7.44)	32 (8.65)	25 (6.79)	25 (6.76)	28 (7.57)
Any ocular disease	436 (29.50)	131 (35.41)	122 (33.15)	97 (26.22)	86 (23.24)

### 3.2 Association between serum carotenoid concentrations and risk of major age-related eye diseases

The association between serum carotenoid concentrations and risk of major eye diseases among middle-aged and older adults is presented in Table 2. After multivariable adjustment, compared to participants in the first quartile (Q1), those in highest quartile (Q4) of serum *α*-carotene (OR: 0.37; 95% CI: 0.21–0.64), *β*-carotene (OR: 0.57; 95% CI: 0.33–0.95), and lutein/zeaxanthin (OR: 0.45; 95% CI: 0.27–0.76) were negatively associated with risk of cataract; those in higher quartile (Q3) of serum *β*-cryptoxanthin (OR: 0.50; 95% CI: 0.27–0.93) was negatively associated with risk of glaucoma; those in highest quartile of serum β-carotene (OR: 0.30; 95% CI: 0.11–0.77) and β-cryptoxanthin (OR: 0.28; 95% CI: 0.12–0.68) were negatively associated with risk of diabetic retinopathy; and those in highest quartile of lycopene (OR: 0.37; 95% CI: 0.180.78) was negatively associated with risk of AMD. In addition, the highest quartile of serum total carotenoid concentrations was negatively associated with risk of cataract (OR: 0.58; 95% CI: 0.35–0.97) compared to the first quartile. Similar results were also observed in univariate analysis of the association between serum carotenoid concentrations and risk of major age-related eye diseases (Supplementary Table 2). Sensitivity analysis, which included additional adjustment for dietary carotenoid intake, showed that the association between serum carotenoids and the risk of age-related eye diseases remained largely unchanged (Supplementary Table 3). In addition, the sensitivity analysis to assess the possible effects of missing data yielded results was also conducted, and the results showed that these associations were robust across sensitivity analyses excluding participants with missing data on covariates (Supplementary Table 4).

**Table 2:** Association between serum carotenoid concentrations and risk of major age-related eye diseases among middle-aged and older adults.

Quartiles of serum carotenoids concentrations	Cataract		Glaucoma		Diabetic retinopathy		Age-related macular degeneration	
	Cases/controls	OR (95% CI)	Cases/controls	OR (95% CI)	Cases/controls	OR (95% CI)	Cases/controls	OR (95% CI)
α-carotene (μmol/L)								
Per 1-SD increase		0.74 (0.58, 0.94)		0.86 (0.64, 1.16)		0.67 (0.40, 1.12)		1.06 (0.85, 1.33)
Quartile 1	61/284	1.00 (Ref)	32/313	1.00 (Ref)	32/313	1.00 (Ref)	25/320	1.00 (Ref)
Quartile 2	59/324	0.62 (0.38, 1.00)	32/351	0.94 (0.54, 1.63)	32/351	1.24 (0.66, 2.35)	27/356	0.73 (0.40, 1.34)
Quartile 3	67/305	0.61 (0.37, 1.00)	30/342	0.77 (0.43, 1.37)	20/352	0.96 (0.46, 2.01)	30/342	0.70 (0.38, 1.30)
Quartile 4	43/335	0.37 (0.21, 0.64)	19/359	0.57 (0.29, 1.09)	10/368	0.43 (0.18, 1.05)	28/350	0.67 (0.35, 1.28)
*P* for trend		<0.001		0.072		0.091		0.263
β-carotene (μmol/L)								
Per 1-SD increase		0.95 (0.80, 1.12)		0.90 (0.70, 1.17)		0.39 (0.20, 0.78)		1.18 (1.01, 1.38)
Quartile 1	55/309	1.00 (Ref)	29/335	1.00 (Ref)	37/327	1.00 (Ref)	20/344	1.00 (Ref)
Quartile 2	52/323	0.62 (0.38, 1.03)	30/345	0.83 (0.47, 1.45)	30/345	1.14 (0.61, 2.12)	34/341	1.44 (0.78, 2.67)
Quartile 3	60/309	0.64 (0.39, 1.06)	23/346	0.57 (0.31, 1.05)	19/350	0.85 (0.42, 1.75)	18/351	0.56 (0.28, 1.14)
Quartile 4	63/307	0.57 (0.33, 0.95)	31/339	0.77 (0.42, 1.40)	8/362	0.30 (0.11, 0.77)	38/332	1.29 (0.68, 2.48)
*P* for trend		0.057		0.269		0.028		0.960
β-cryptoxanthin (μmol/L)								
Per 1-SD increase		0.88 (0.70, 1.10)		1.02 (0.82, 1.28)		0.56 (0.37, 0.86)		0.83 (0.61, 1.12)
Quartile 1	61/309	1.00 (Ref)	35/335	1.00 (Ref)	32/338	1.00 (Ref)	28/342	1.00 (Ref)
Quartile 2	60/308	1.08 (0.67, 1.72)	28/340	0.76 (0.44, 1.31)	32/336	0.98 (0.52, 1.87)	22/346	0.80 (0.43, 1.47)
Quartile 3	63/307	1.13 (0.70, 1.83)	19/351	0.50 (0.27, 0.93)	17/353	0.61 (0.29, 1.31)	39/331	1.34 (0.76, 2.35)
Quartile 4	46/324	0.69 (0.41, 1.16)	31/339	0.88 (0.49, 1.55)	13/357	0.28 (0.12, 0.68)	21/349	0.68 (0.36, 1.31)
*P* for trend		0.236		0.415		0.004		0.620
Lycopene (μmol/L)								
Per 1-SD increase		0.99 (0.81, 1.21)		0.94 (0.76, 1.18)		0.99 (0.76, 1.30)		0.74 (0.57, 0.95)
Quartile 1	83/286	1.00 (Ref)	41/328	1.00 (Ref)	29/340	1.00 (Ref)	35/334	1.00 (Ref)
Quartile 2	62/307	0.83 (0.54, 1.28)	26/343	0.62 (0.36, 1.06)	22/347	0.90 (0.45, 1.80)	35/334	1.08 (0.64, 1.83)
Quartile 3	50/320	0.97 (0.61, 1.55)	25/345	0.67 (0.39, 1.17)	25/345	1.14 (0.57, 2.26)	29/341	0.96 (0.54, 1.69)
Quartile 4	35/335	0.83 (0.50, 1.39)	21/349	0.66 (0.37, 1.20)	18/352	0.99 (0.47, 2.08)	11/359	0.37 (0.18, 0.78)
*P* for trend		0.605		0.163		0.857		0.019
Lutein/zeaxanthin (μmol/L)								
Per 1-SD increase		0.81 (0.67, 0.98)		0.97 (0.78, 1.21)		1.04 (0.81, 1.32)		1.29 (1.08, 1.55)
Quartile 1	71/299	1.00 (Ref)	25/345	1.00 (Ref)	32/338	1.00 (Ref)	25/345	1.00 (Ref)
Quartile 2	65/298	0.91 (0.58, 1.45)	26/337	1.03 (0.57, 1.86)	21/342	0.64 (0.31, 1.31)	32/331	1.27 (0.71, 2.29)
Quartile 3	59/313	0.81 (0.50, 1.30)	37/335	1.41 (0.79, 2.50)	20/352	0.76 (0.37, 1.60)	28/344	1.16 (0.63, 2.13)
Quartile 4	35/338	0.45 (0.27, 0.76)	25/348	0.95 (0.51, 1.76)	21/352	0.64 (0.31, 1.34)	25/348	1.09 (0.58, 2.05)
*P* for trend		0.004		0.860		0.326		0.893
Total carotenoid (μmol/L)								
Per 1-SD increase		0.88 (0.73, 1.07)		0.91 (0.72, 1.16)		0.73 (0.51, 1.05)		1.10 (0.90, 1.35)
Quartile 1	76/294	1.00 (Ref)	35/335	1.00 (Ref)	33/337	1.00 (Ref)	32/338	1.00 (Ref)
Quartile 2	63/305	0.98 (0.62, 1.53)	31/337	0.85 (0.50, 1.47)	29/339	1.08 (0.56, 2.08)	25/343	0.81 (0.45, 1.45)
Quartile 3	48/322	0.59 (0.37, 0.96)	22/348	0.58 (0.32, 1.05)	19/351	1.07 (0.52, 2.19)	25/345	0.71 (0.39, 1.28)
Quartile 4	43/327	0.58 (0.35, 0.97)	25/345	0.77 (0.43, 1.40)	13/357	0.54 (0.24, 1.22)	28/342	0.87 (0.48, 1.59)
*P* for trend		0.010		0.211		0.214		0.569

P for trend was obtained by including the quartile number as a continuous variable in the regression model. Models were adjusted for age (continuous), sex (male or female), race/ethnicity (non-Hispanic white, black, Mexican-American, other Hispanic, or other race/ethnicity), education level (less than high school, high school or equivalent, or college or above), family income-to-poverty ratio (<1.3, 1.3 to ≤3.5, >3.5, or missing), body mass index (<18.5, 18.5 to <25, 25 to <30, ≥30 kg/m^2^, or missing), drinking status (non-drinker or drinker), smoking status (never smoker, former smoker, current smoker, or missing), HbA1c (<6.5%, ≥6.5%, or missing), hypertension (yes, no, or missing), hypercholesterolemia (yes, no, or missing), and cancer (yes, no, or missing).

### 3.3 Association between serum carotenoid concentrations and risk of any ocular disease among middle-aged and older adults

**Figure 1:**
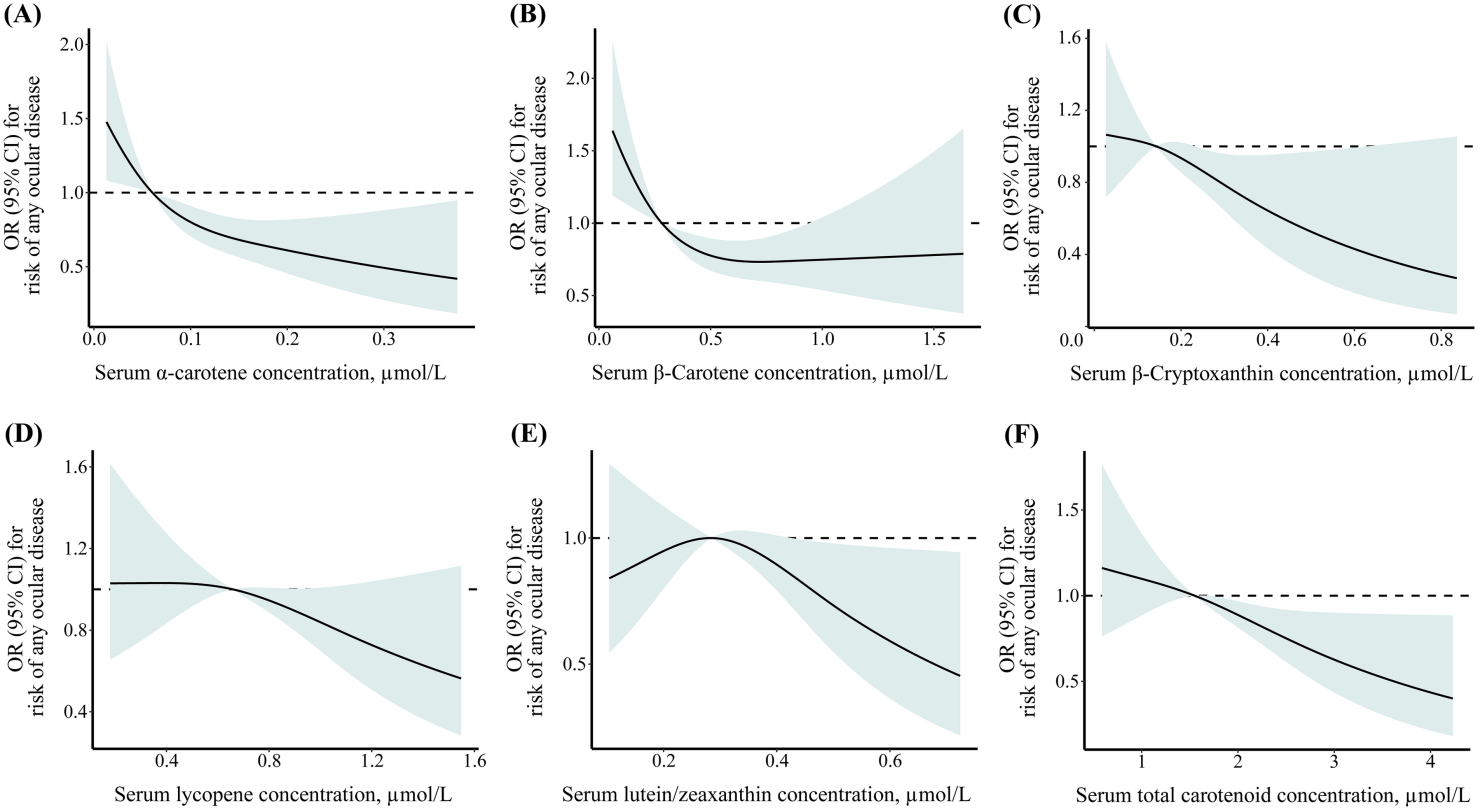
Dose–response association between geriatric nutritional risk index and cognitive function level among older adults with cardiometabolic disease. **(A)** α-carotene; **(B)** β-carotene; **(C)** β-cryptoxanthin; **(D)** lycopene; **(E)** lutein/zeaxanthin; **(F)** total carotenoid.

As shown in Figure 1, the restricted cubic spline results showed that the negative dose–response association between serum *α*-carotene, *β*-carotene, β-cryptoxanthin, lycopene, lutein/zeaxanthin, and total carotenoid concentrations and risk of any ocular disease among middle-aged and older adults. In addition, multivariable adjustment model results showed that compared to participants in the first quartile, those in highest quartile of serum *α*-carotene (OR: 0.47; 95% CI: 0.31–0.71), *β*-carotene (OR: 0.54; 95% CI: 0.36–0.81), β-cryptoxanthin (OR: 0.67; 95% CI: 0.45–0.99), lycopene (OR: 0.62; 95% CI: 0.42–0.91), and total carotenoid (OR: 0.63; 95% CI: 0.42–0.92) concentrations were negatively associated with risk of any ocular disease (Table 3). The associations between serum carotenoid concentrations and risk of any ocular disease were also robust across sensitivity analyses excluding participants with missing data on covariates (Supplementary Table 5).

**Table 3:** Association between serum carotenoid concentrations and risk of ocular disease among middle-aged and older adults.

	Quartiles of serum carotenoid concentrations (μmol/L)				Per 1-SD increase	*P* for trend
	Quartile 1	Quartile 2	Quartile 3	Quartile 4		
	OR (95% CI)	OR (95% CI)	OR (95% CI)	OR (95% CI)		
α-carotene						
Cases/controls	112/233	126/257	115/257	83/295		
Crude model	1.00 (Ref)	1.02 (0.75, 1.39)	0.93 (0.68, 1.28)	0.59 (0.42, 0.82)	0.85 (0.74, 0.97)	0.002
Multivariate model	1.00 (Ref)	0.96 (0.67, 1.38)	0.73 (0.50, 1.08)	0.47 (0.31, 0.71)	0.81 (0.68, 0.97)	<0.001
β-carotene						
Cases/controls	116/248	115/260	100/269	105/265		
Crude model	1.00 (Ref)	0.95 (0.69, 1.29)	0.79 (0.58, 1.09)	0.85 (0.62, 1.16)	1.02 (0.92, 1.14)	0.188
Multivariate model	1.00 (Ref)	0.81 (0.56, 1.16)	0.54 (0.37, 0.80)	0.54 (0.36, 0.81)	0.96 (0.84, 1.09)	<0.001
*β*-cryptoxanthin						
Cases/controls	118/252	111/257	115/255	92/278		
Crude model	1.00 (Ref)	0.92 (0.68, 1.26)	0.96 (0.71, 1.31)	0.71 (0.51, 0.97)	0.89 (0.78, 1.01)	0.057
Multivariate model	1.00 (Ref)	0.94 (0.66, 1.35)	1.02 (0.70, 1.48)	0.67 (0.45, 0.99)	0.90 (0.77, 1.06)	0.085
Lycopene						
Cases/controls	143/226	113/256	110/260	70/300		
Crude model	1.00 (Ref)	0.70 (0.51, 0.95)	0.67 (0.49, 0.91)	0.37 (0.26, 0.51)	0.70 (0.62, 0.80)	<0.001
Multivariate model	1.00 (Ref)	0.80 (0.56, 1.13)	0.98 (0.69, 1.39)	0.62 (0.42, 0.91)	0.90 (0.78, 1.04)	0.057
Lutein/zeaxanthin						
Cases/controls	115/255	114/249	118/254	89/284		
Crude model	1.00 (Ref)	1.01 (0.74, 1.38)	1.04 (0.76, 1.42)	0.69 (0.50, 0.96)	0.99 (0.89, 1.11)	0.046
Multivariate model	1.00 (Ref)	0.99 (0.69, 1.43)	1.06 (0.73, 1.54)	0.69 (0.47, 1.01)	1.01 (0.88, 1.15)	0.086
Total carotenoid						
Cases/controls	131/239	122/246	97/273	86/284		
Crude model	1.00 (Ref)	0.90 (0.67, 1.23)	0.65 (0.47, 0.89)	0.55 (0.40, 0.76)	0.86 (0.76, 0.97)	<0.001
Multivariate model	1.00 (Ref)	1.05 (0.73, 1.49)	0.70 (0.49, 1.02)	0.63 (0.42, 0.92)	0.91 (0.78, 1.04)	0.004

P for trend was obtained by including the quartile number as a continuous variable in the regression model. Models were adjusted for age (continuous), sex (male or female), race/ethnicity (non-Hispanic white, black, Mexican-American, other Hispanic, or other race/ethnicity), education level (less than high school, high school or equivalent, college or above, or missing), family income-to-poverty ratio (<1.3, 1.3 to ≤3.5, >3.5, or missing), body mass index (<18.5, 18.5 to <25, 25 to <30, ≥30 kg/m2, or missing), drinking status (non-drinker or drinker), smoking status (never smoker, former smoker, current smoker, or missing), HbA1c (<6.5%, ≥6.5%, or missing), hypertension (yes, no, or missing), hypercholesterolemia (yes, no, or missing), and cancer (yes, no, or missing).

### 3.4 Subgroup analysis

Subgroup analysis stratified by sex was conducted to examine the association between serum carotenoid concentrations and risk of any ocular disease (Table 4). Compared to participants in the first quartile, those in highest quartile of serum *α*-carotene (OR: 0.16; 95% CI: 0.08–0.31), *β*-carotene (OR: 0.39; 95% CI: 0.21–0.75), β-cryptoxanthin (OR: 0.46; 95% CI: 0.24–0.87), lycopene (OR: 0.45; 95% CI: 0.24–0.83), lutein/zeaxanthin (OR: 0.43; 95% CI: 0.23–0.78), and total carotenoid (OR: 0.41; 95% CI: 0.22–0.76) concentrations were negatively associated with risk of any ocular disease among female participants. By contrast, no association was observed between serum total carotenoid concentrations and risk of any ocular disease among male participants (all *P* for trend>0.05).

**Table 4:** Association between serum carotenoid concentrations and risk of ocular disease among middle-aged and older adults by sex.

Quartiles of serum carotenoid concentrations	Male		Female		*P* for interaction
	Cases/controls	OR (95% CI)	Cases/controls	OR (95% CI)	
α-carotene					< 0.001
Per 1-SD increase		1.09 (0.88, 1.35)		0.59 (0.44, 0.79)	
Quartile 1	59/156	1.00 (Ref)	53/77	1.00 (Ref)	
Quartile 2	75/136	1.47 (0.91, 2.36)	51/121	0.45 (0.25, 0.84)	
Quartile 3	53/137	0.85 (0.51, 1.43)	62/120	0.46 (0.25, 0.86)	
Quartile 4	44/115	1.01 (0.58, 1.74)	39/180	0.16 (0.08, 0.31)	
*P* for trend		0.518		<0.001	
β-carotene					0.318
Per 1-SD increase		1.05 (0.84, 1.32)		0.91 (0.76, 1.08)	
Quartile 1	75/166	1.00 (Ref)	41/82	1.00 (Ref)	
Quartile 2	64/139	0.93 (0.58, 1.48)	51/121	0.58 (0.31, 1.08)	
Quartile 3	51/142	0.61 (0.37, 0.99)	49/127	0.39 (0.20, 0.74)	
Quartile 4	41/97	0.66 (0.38, 1.13)	64/168	0.39 (0.21, 0.75)	
P for trend		0.044		<0.001	
β-cryptoxanthin					0.028
Per 1-SD increase		1.05 (0.88, 1.27)		0.73 (0.56, 0.96)	
Quartile 1	65/152	1.00 (Ref)	53/100	1.00 (Ref)	
Quartile 2	57/136	0.94 (0.58, 1.52)	54/121	0.90 (0.50, 1.60)	
Quartile 3	62/129	1.08 (0.66, 1.75)	53/126	0.95 (0.52, 1.74)	
Quartile 4	47/127	0.90 (0.53, 1.51)	45/151	0.46 (0.24, 0.87)	
*P* for trend		0.843		0.025	
Lycopene					0.086
Per 1-SD increase		1.01 (0.84, 1.22)		0.78 (0.61, 0.99)	
Quartile 1	69/130	1.00 (Ref)	74/96	1.00 (Ref)	
Quartile 2	57/126	0.92 (0.57, 1.49)	56/130	0.70 (0.41, 1.18)	
Quartile 3	63/133	1.28 (0.79, 2.09)	47/127	0.71 (0.41, 1.23)	
Quartile 4	42/155	0.88 (0.52, 1.48)	28/145	0.45 (0.24, 0.83)	
*P* for trend		0.981		0.017	
Lutein/zeaxanthin					0.008
Per 1-SD increase		1.15 (0.98, 1.36)		0.79 (0.63, 0.99)	
Quartile 1	56/138	1.00 (Ref)	59/117	1.00 (Ref)	
Quartile 2	61/144	1.04 (0.63, 1.73)	54/109	0.80 (0.45, 1.44)	
Quartile 3	65/128	1.34 (0.81, 2.21)	52/122	0.70 (0.39, 1.26)	
Quartile 4	49/134	0.91 (0.54, 1.53)	40/150	0.43 (0.23, 0.78)	
*P* for trend		0.974		0.006	
Total carotenoid					0.015
Per 1-SD increase		1.09 (0.89, 1.33)		0.75 (0.60, 0.94)	
Quartile 1	75/144	1.00 (Ref)	56/95	1.00 (Ref)	
Quartile 2	60/133	0.93 (0.58, 1.49)	62/113	1.13 (0.64, 1.98)	
Quartile 3	51/147	0.76 (0.47, 1.23)	46/126	0.55 (0.30, 1.00)	
Quartile 4	45/120	0.90 (0.53, 1.51)	41/164	0.41 (0.22, 0.76)	
*P* for trend		0.484		<0.001	

P for trend was obtained by including the quartile number as a continuous variable in the regression model. P for interaction was calculated using the likelihood ratio test. Models were adjusted for age (continuous), race/ethnicity (non-Hispanic white, black, Mexican-American, other Hispanic, or other race/ethnicity), education level (less than high school, high school or equivalent, college or above, or missing), family income-to-poverty ratio (<1.3, 1.3 to ≤3.5, >3.5, or missing), body mass index (<18.5, 18.5 to <25, 25 to <30, ≥30 kg/m2, or missing), drinking status (non-drinker or drinker), smoking status (never smoker, former smoker, current smoker, or missing), HbA1c (<6.5%, ≥6.5%, or missing), hypertension (yes, no, or missing), hypercholesterolemia (yes, no, or missing), and cancer (yes, no, or missing).

Furthermore, compared to those in the first quartile, the highest quartile of serum *α*-carotene (OR: 0.15; 95% CI: 0.06–0.36), *β*-carotene (OR: 0.42; 95% CI: 0.18–0.97), lutein/zeaxanthin (OR: 0.26; 95% CI: 0.11–0.61), and total carotenoid (OR: 0.35; 95% CI: 0.15–0.80) concentrations were negatively associated with risk of cataract among female participants (Supplementary Table 6); the highest quartile of serum *α*-carotene (OR: 0.24; 95% CI: 0.09–0.67) was negatively associated with risk of glaucoma among female participants (Supplementary Table 7); those in highest quartile of serum α-carotene (OR: 0.22; 95% CI: 0.06–0.82) and *β*-carotene (OR: 0.24; 95% CI: 0.06–0.95) were negatively associated with risk of diabetic retinopathy among female participants; those in highest quartile of serum β-cryptoxanthin (OR: 0.25; 95% CI: 0.07–0.88) was also negatively associated with risk of diabetic retinopathy among male participants (Supplementary Table 8); and those in the highest quartile of serum lycopene (OR: 0.14; 95% CI: 0.03, 0.67) was negatively associated with risk of AMD among female participants (Supplementary Table 9). In addition, we performed stratified analyses by smoking status, and did not find a statistically significant interaction between smoking status and *β*-carotene, β-cryptoxanthin, lutein/zeaxanthin, lycopene, and total carotenoid levels on the risk of age-related ocular diseases in our analysis (Supplementary Table 10).

## 4 Discussion

The present study investigated the associations between the serum carotenoid concentration and the risk of major age-related eye diseases in a nationally representative sample of the middle-aged and older adult population in the United States. The findings indicated that higher serum concentrations of *α*-carotene, *β*-carotene, and lutein/zeaxanthin were negatively associated with the risk of cataracts; a higher serum concentration of β-cryptoxanthin was negatively associated with the risk of glaucoma; higher serum concentrations of *β*-carotene and β-cryptoxanthin were negatively associated with the risk of diabetic retinopathy; and a higher serum concentration of lycopene was negatively associated with the risk of AMD. In addition, we discovered that serum concentrations of *α*-carotene, β-carotene, β-cryptoxanthin, lycopene, and total carotenoids were inversely associated with the risk of any ocular disease, particularly among the female participants of the present study.

Although the causes of age-related eye diseases are complex and multifactorial, oxidative stress is widely recognized as a common factor that contributes to the development of age-related eye diseases in middle-aged and older adults (31, 32). The eyes are particularly vulnerable to oxidative stress due to factors such as direct exposure to ultraviolet light, a high content of mitochondria, and high metabolic activity (2, 33). Studies have demonstrated that reactive oxygen species (ROS) can cause cataracts by damaging cell membrane fibers and crystalline proteins (34). The complex non-enzymatic and enzymatic antioxidant system in the lens can scavenge ROS for preserving lens proteins, whereas the weakening of this antioxidant defense system can cause damage to lens molecules and disrupt their repair mechanisms (35). Furthermore, in several studies examining the superoxide dismutase-knockdown mouse model, mice with lower levels of antioxidants were reported to exhibit higher ROS levels; these mice also displayed various AMD characteristics, including drusen, thickened Bruch’s membrane, and choroidal neovascularization (36–38). Oxidative stress has been speculated to be involved in diabetic retinopathy. For example, studies have demonstrated that oxidative stress can alter the blood–retinal barrier and increase vascular permeability, which are the most prominent characteristics of diabetic retinopathy (39). Therefore, antioxidants may alleviate diabetic retinopathy. A study reported that the long-term administration of antioxidants inhibited the development of early-stage diabetic retinopathy in diabetic rats (40).

A growing body of evidence suggests that carotenoids have antioxidant effects and that higher circulating concentrations of carotenoids are associated with lower risks of various oxidative stress–related chronic diseases, such as diabetes, hypertension, and non-alcoholic fatty liver disease (11, 26, 41, 42). Because carotenoids may be involved in cellular signaling pathways related to inflammation and oxidative stress, they may inhibit oxidative stress and inflammation (43). However, to date, no systematic evaluation has examined the associations between the serum concentrations of various carotenoids and the development of age-related eye diseases, and most studies have mainly focused on single eye diseases. A meta-analysis of observational studies indicated that the serum concentration of *β*-carotene was associated with a reduced risk of cataracts (44). Similarly, inverse associations were observed between the serum concentrations of *α*-carotene, lutein, and zeaxanthin and the risk of cataracts (45), corresponding to our findings. To the best of our knowledge, no study has evaluated the associations between serum concentrations of carotenoids and the risk of glaucoma. Only one study reported that the dietary intake of *α*-carotene, *β*-carotene, or lutein/zeaxanthin was associated with a decreased risk of glaucoma among older African-American women. The present study also identified negative associations between the serum concentration of *α*-carotene and the risk of glaucoma among female participants (46). Regarding the associations between the serum concentrations of carotenoids and the risk of diabetic retinopathy, a study reported that the plasma levels of carotenoids, including α-carotene, *β*-carotene, β-cryptoxanthin, lycopene, and lutein/zeaxanthin, were lower in individuals with diabetic retinopathy than in those without diabetic retinopathy (47). Similar to our findings, the results of a cross-sectional study conducted in a Chinese population indicated that a higher serum concentration of *β*-carotene is associated with a lower risk of diabetic retinopathy, suggesting that β-carotene plays a protective role against diabetic retinopathy (23). Furthermore, in a matched case–control study of 164 patients with AMD, higher concentrations of carotenoids, including β-carotene, *β*-cryptoxanthin, lycopene, and lutein/zeaxanthin, were revealed to be inversely associated with the risk of AMD (48). Our study also revealed an inverse association between the serum concentration of lycopene and the risk of AMD.

The results of the present study suggested that serum concentrations of *α*-carotene, *β*-carotene, β-cryptoxanthin, lycopene, lutein/zeaxanthin, and total carotenoids were associated with a decreased risk of any ocular disease among the female participants. By contrast, no association was identified between the serum concentrations of carotenoids and the risk of any ocular disease among the male participants. While this finding is intriguing, the underlying mechanisms remain uncertain and should be interpreted with caution. A possible explanation for this phenomenon is that relative to men, women are typically more sensitive to oxidative stress and tend to exhibit higher levels of oxidative stress (49–51). In particular, for postmenopausal women, the lack of estrogen often leads to increased oxidative stress (52). In addition, women may have a higher risk of age-related eye diseases than men because of various socioeconomic factors and the effects of estrogen withdrawal during menopause (4). In addition, the stronger inverse associations observed in women may reflect estrogen’s modulation of carotenoid metabolism and absorption. For instance, scavenger receptor class B member 1 (SR-B1) is a multiligand receptor that facilitates the uptake of cholesteryl esters from high-density lipoproteins (HDLs) and the transport of carotenoids. Its expression is transcriptionally upregulated by estrogen through the direct binding of estrogen receptors to estrogen response elements (EREs) in its promoter (53). This is supported by our supplementary analysis showing lower carotenoid levels in female participants compared to male participants (54). However, it is important to note that these explanations remain hypothetical. Residual confounding by unmeasured socioeconomic or lifestyle factors. Therefore, our results should be seen as generating a hypothesis that warrants further investigation in studies designed specifically to explore causal mechanisms behind sex differences in carotenoid metabolism and ocular protection.

The present study has several strengths. First, we comprehensively assessed the associations between the serum concentrations of various carotenoids and the risk of various age-related eye diseases, including cataract, glaucoma, diabetic retinopathy, and AMD. Notably, our study is the first to explore the dose–response relationship between the serum concentrations of various carotenoids and the risk of any ocular disease. Second, because we used data from the National Health and Nutrition Examination Survey, which is a nationally representative database, our findings can be generalized to the community-dwelling population in the United States. Third, our analyses were adjusted for an extensive set of confounders, including socioeconomic and health factors. Finally, serum carotenoid levels provide a more robust and biologically relevant measure than dietary intake estimates for studying age-related eye diseases. Unlike questionnaire-based dietary data, serum concentrations objectively reflect bioavailable carotenoid exposure, integrating absorption efficiency, metabolic variation, and contributions from both diet and supplements. Crucially, they exhibit stronger physiological plausibility for antioxidant protection in ocular tissues and, in our analyses, remained independently associated with disease risk even after adjustment for dietary intake, highlighting their value as a superior biomarker in nutritional ophthalmology research.

Nonetheless, the present study still has several limitations. First, because of the cross-sectional design of our study, we could not explore the causal relationship between the serum concentrations of the studied carotenoids and the development of age-related eye diseases. Second, the cohort consisted predominantly of non-Hispanic White participants, with underrepresented groups comprising a smaller proportion, which limits the generalizability of our findings. Third, our study is limited by the classification of diabetic retinopathy within the NHANES database, which did not separately identify proliferative diabetic retinopathy (PDR). Consequently, our analysis was restricted to non-proliferative stages, potentially diluting the observed effect sizes and preventing an assessment of whether higher carotenoid levels are associated with a reduced risk of progressing to sight-threatening PDR. This may result in a conservative underestimation of the true protective association. Fourth, cataract and glaucoma were defined based on self-reported data obtained during a personal interview. This method does not allow differentiation between primary open-angle glaucoma (POAG) and ocular hypertension (OHT). Since OHT is not associated with the optic nerve damage characteristic of POAG, this misclassification likely biases the observed associations toward null, thereby potentially underestimating the relationship between carotenoid levels and neurodegenerative glaucomatous pathology. Future prospective studies should incorporate detailed clinical assessments, such as optic nerve head imaging, visual field testing, to improve diagnostic accuracy and better distinguish between glaucoma subtypes. Fifth, due to constraints in the NHANES dataset, we did not exclude participants with non-age-related ocular conditions (e.g., uveitis and traumatic cataract) or a history of cataract surgery, which may introduce some potential for confounding. Furthermore, the findings of the present study could have been influenced by residual confounders, such as dietary supplementation (AREDS formulations, multivitamins) and medication use (statins, corticosteroids). Finally, the sample size of the present study was small because limited data were available, and the associations between the serum concentrations of several carotenoids and the development of age-related eye diseases approached statistical significance. Although the FDR correction was applied to mitigate the risk of false positives resulting from multiple comparisons, some significant findings may still be attributable to type I error. Therefore, our overall findings must be further verified through prospective studies with larger sample sizes.

## 5 Conclusion

Our study demonstrated that higher serum concentrations of carotenoids, particularly *α*-carotene, *β*-carotene, β-cryptoxanthin, and lycopene, were associated with a lower risk of age-related eye diseases. The present findings need to be verified in prospective studies with a larger sample size.

## Data Availability

Publicly available datasets were analyzed in this study. This data can be found at: https://www.cdc.gov/nchs/nhanes/.

## References

[1] Wong WL Su X Li X Cheung CM Klein R Cheng CY . Global prevalence of age-related macular degeneration and disease burden projection for 2020 and 2040: a systematic review and meta-analysis. Lancet Glob Health. (2014) 2:e106–16. doi: 10.1016/s2214-109x(13)70145-1 25104651

[2] Bungau S Abdel-Daim MM Tit DM Ghanem E Sato S Maruyama-Inoue M . Health benefits of polyphenols and carotenoids in age-related eye diseases. Oxidative Med Cell Longev. (2019) 2019:1–22. doi: 10.1155/2019/9783429, PMID: 30891116 PMC6390265

[3] Rauf A Imran M Suleria HAR Ahmad B Peters DG Mubarak MS. A comprehensive review of the health perspectives of resveratrol. Food Funct. (2017) 8:4284–305. doi: 10.1039/c7fo01300k, PMID: 29044265

[4] Zetterberg M. Age-related eye disease and gender. Maturitas. (2016) 83:19–26. doi: 10.1016/j.maturitas.2015.10.005, PMID: 26508081

[5] Voleti VB Hubschman JP. Age-related eye disease. Maturitas. (2013) 75:29–33. doi: 10.1016/j.maturitas.2013.01.018, PMID: 23474322

[6] Ehrlich JR Ramke J Macleod D Burn H Lee CN Zhang JH . Association between vision impairment and mortality: a systematic review and meta-analysis. Lancet Glob Health. (2021) 9:e418–30. doi: 10.1016/s2214-109x(20)30549-0, PMID: 33607015 PMC7966688

[7] Ghanbarnia MJ Hosseini SR Ghasemi M Roustaei GA Mekaniki E Ghadimi R . Association of age-related eye diseases with cognitive frailty in older adults: a population-based study. Aging Clin Exp Res. (2023) 35:1731–40. doi: 10.1007/s40520-023-02458-z, PMID: 37269465

[8] Knudtson MD Klein BE Klein R. Age-related eye disease, visual impairment, and survival: the beaver dam eye study. Arch Ophthalmol. (2006) 124:243–9. doi: 10.1001/archopht.124.2.243, PMID: 16476894

[9] Karakuş MM Çalışkan UK. Phytotherapeutic and natural compound applications for age-related, inflammatory and serious eye ailments. Curr Mol Pharmacol. (2021) 14:689–713. doi: 10.2174/1874467213666201221163210, PMID: 33349225

[10] Rodriguez-Concepcion M Avalos J Bonet ML Boronat A Gomez-Gomez L Hornero-Mendez D . A global perspective on carotenoids: metabolism, biotechnology, and benefits for nutrition and health. Prog Lipid Res. (2018) 70:62–93. doi: 10.1016/j.plipres.2018.04.004, PMID: 29679619

[11] Xiao ML Chen GD Zeng FF Qiu R Shi WQ Lin JS . Higher serum carotenoids associated with improvement of non-alcoholic fatty liver disease in adults: a prospective study. Eur J Nutr. (2019) 58:721–30. doi: 10.1007/s00394-018-1678-1, PMID: 29594435

[12] Qiu Z Chen X Geng T Wan Z Lu Q Li L . Associations of serum carotenoids with risk of cardiovascular mortality among individuals with type 2 diabetes: results from NHANES. Diabetes Care. (2022) 45:1453–61. doi: 10.2337/dc21-2371, PMID: 35503926

[13] Johra FT Bepari AK Bristy AT Reza HM. A mechanistic review of β-carotene, lutein, and zeaxanthin in eye health and disease. Antioxidants. (2020) 9:1046. doi: 10.3390/antiox9111046, PMID: 33114699 PMC7692753

[14] Widjaja-Adhi MAK Ramkumar S von Lintig J. Protective role of carotenoids in the visual cycle. FASEB J. (2018) 32:6305–15. doi: 10.1096/fj.201800467R, PMID: 29882710 PMC6181638

[15] Lem DW Gierhart DL Davey PG. A systematic review of carotenoids in the management of diabetic retinopathy. Nutrients. (2021) 13:2441. doi: 10.3390/nu13072441, PMID: 34371951 PMC8308772

[16] Brown L Rimm EB Seddon JM Giovannucci EL Chasan-Taber L Spiegelman D . A prospective study of carotenoid intake and risk of cataract extraction in US men. Am J Clin Nutr. (1999) 70:517–24. doi: 10.1093/ajcn/70.4.517, PMID: 10500021

[17] Jiang H Yin Y Wu CR Liu Y Guo F Li M . Dietary vitamin and carotenoid intake and risk of age-related cataract. Am J Clin Nutr. (2019) 109:43–54. doi: 10.1093/ajcn/nqy270, PMID: 30624584

[18] Loughman J Loskutova E Butler JS Siah WF O'Brien C. Macular pigment response to lutein, zeaxanthin, and meso-zeaxanthin supplementation in open-angle glaucoma: a randomized controlled trial. Ophthalmol Sci. (2021) 1:100039. doi: 10.1016/j.xops.2021.100039, PMID: 36247822 PMC9562333

[19] Chew EY Clemons TE Agrón E Domalpally A Keenan TDL Vitale S . Long-term outcomes of adding lutein/zeaxanthin and ω-3 fatty acids to the AREDS supplements on age-related macular degeneration progression: AREDS2 report 28. JAMA Ophthalmol. (2022) 140:692–8. doi: 10.1001/jamaophthalmol.2022.1640, PMID: 35653117 PMC9164119

[20] Satia JA Littman A Slatore CG Galanko JA White E. Long-term use of beta-carotene, retinol, lycopene, and lutein supplements and lung cancer risk: results from the VITamins and lifestyle (VITAL) study. Am J Epidemiol. (2009) 169:815–28. doi: 10.1093/aje/kwn409, PMID: 19208726 PMC2842198

[21] Zhang J Xiao L Zhao X Wang P Yang C. Exploring the association between composite dietary antioxidant index and ocular diseases: a cross-sectional study. BMC Public Health. (2025) 25:625. doi: 10.1186/s12889-025-21867-5, PMID: 39953504 PMC11829354

[22] Xiong R Yuan Y Zhu Z Wu Y Ha J Han X . Micronutrients and diabetic retinopathy: evidence from the National Health and nutrition examination survey and a meta-analysis. Am J Ophthalmol. (2022) 238:141–56. doi: 10.1016/j.ajo.2022.01.005, PMID: 35033539

[23] She C Shang F Zhou K Liu N. Serum carotenoids and risks of diabetes and diabetic retinopathy in a Chinese population sample. Curr Mol Med. (2017) 17:287–97. doi: 10.2174/1566524017666171106112131, PMID: 29110607

[24] Dherani M Murthy GV Gupta SK Young IS Maraini G Camparini M . Blood levels of vitamin C, carotenoids and retinol are inversely associated with cataract in a north Indian population. Invest Ophthalmol Vis Sci. (2008) 49:3328–35. doi: 10.1167/iovs.07-1202, PMID: 18421094

[25] Zhou H Zhao X Johnson EJ Lim A Sun E Yu J . Serum carotenoids and risk of age-related macular degeneration in a Chinese population sample. Invest Ophthalmol Vis Sci. (2011) 52:4338–44. doi: 10.1167/iovs.10-6519, PMID: 21508112

[26] Zhu X Shi M Pang H Cheang I Zhu Q Guo Q . Inverse association of serum carotenoid levels with prevalence of hypertension in the general adult population. Front Nutr. (2022) 9:971879. doi: 10.3389/fnut.2022.971879, PMID: 36245540 PMC9563225

[27] Li HY Yang Q Dong L Zhang RH Zhou WD Wu HT . Visual impairment and major eye diseases in stroke: a national cross-sectional study. Eye (Lond). (2023) 37:1850–5. doi: 10.1038/s41433-022-02238-5, PMID: 36131090 PMC10275905

[28] Diagnosis and classification of diabetes mellitus. Diabetes Care. (2011) 34 Suppl 1:S62–9. doi: 10.2337/dc11-S062, PMID: 21193628 PMC3006051

[29] Jaddoe VW de Jonge LL Hofman A Franco OH Steegers EA Gaillard R. First trimester fetal growth restriction and cardiovascular risk factors in school age children: population based cohort study. BMJ (Clinical research ed). (2014) 348:g14. doi: 10.1136/bmj.g14, PMID: 24458585 PMC3901421

[30] Kernan WN Viscoli CM Brass LM Broderick JP Brott T Feldmann E . Phenylpropanolamine and the risk of hemorrhagic stroke. N Engl J Med. (2000) 343:1826–32. doi: 10.1056/nejm200012213432501, PMID: 11117973

[31] Choo PP Woi PJ Bastion MC Omar R Mustapha M Md Din N. Review of evidence for the usage of antioxidants for eye aging. Biomed Res Int. (2022) 2022:5810373. doi: 10.1155/2022/5810373, PMID: 36225983 PMC9550496

[32] Blasiak J Sobczuk P Pawlowska E Kaarniranta K. Interplay between aging and other factors of the pathogenesis of age-related macular degeneration. Ageing Res Rev. (2022) 81:101735. doi: 10.1016/j.arr.2022.101735, PMID: 36113764

[33] Shoham A Hadziahmetovic M Dunaief JL Mydlarski MB Schipper HM. Oxidative stress in diseases of the human cornea. Free Radic Biol Med. (2008) 45:1047–55. doi: 10.1016/j.freeradbiomed.2008.07.021, PMID: 18718524

[34] Spector A. Oxidative stress-induced cataract: mechanism of action. FASEB J. (1995) 9:1173–82. doi: 10.1096/fasebj.9.12.7672510, PMID: 7672510

[35] Heruye SH Maffofou Nkenyi LN Singh NU Yalzadeh D Ngele KK Njie-Mbye YF . Current trends in the pharmacotherapy of cataracts. Pharmaceuticals (Basel, Switzerland). (2020) 13:15. doi: 10.3390/ph13010015, PMID: 31963166 PMC7168925

[36] Imamura Y Noda S Hashizume K Shinoda K Yamaguchi M Uchiyama S . Drusen, choroidal neovascularization, and retinal pigment epithelium dysfunction in SOD1-deficient mice: a model of age-related macular degeneration. Proc Natl Acad Sci USA. (2006) 103:11282–7. doi: 10.1073/pnas.0602131103, PMID: 16844785 PMC1544079

[37] Justilien V Pang JJ Renganathan K Zhan X Crabb JW Kim SR . SOD2 knockdown mouse model of early AMD. Invest Ophthalmol Vis Sci. (2007) 48:4407–20. doi: 10.1167/iovs.07-0432, PMID: 17898259 PMC6549721

[38] Goodman D Ness S. The role of oxidative stress in the aging eye. Life (Basel, Switzerland). (2023) 13:837. doi: 10.3390/life13030837, PMID: 36983992 PMC10052045

[39] Frey T Antonetti DA. Alterations to the blood-retinal barrier in diabetes: cytokines and reactive oxygen species. Antioxid Redox Signal. (2011) 15:1271–84. doi: 10.1089/ars.2011.3906, PMID: 21294655

[40] Kowluru RA Tang J Kern TS. Abnormalities of retinal metabolism in diabetes and experimental galactosemia. VII. Effect of long-term administration of antioxidants on the development of retinopathy. Diabetes. (2001) 50:1938–42. doi: 10.2337/diabetes.50.8.1938, PMID: 11473058

[41] Zhu R Chen B Bai Y Miao T Rui L Zhang H . Lycopene in protection against obesity and diabetes: a mechanistic review. Pharmacol Res. (2020) 159:104966. doi: 10.1016/j.phrs.2020.104966, PMID: 32535223

[42] Wang M Tang R Zhou R Qian Y Di D. The protective effect of serum carotenoids on cardiovascular disease: a cross-sectional study from the general US adult population. Front Nutr. (2023) 10:1154239. doi: 10.3389/fnut.2023.1154239, PMID: 37502714 PMC10368866

[43] Yao Y Goh HM Kim JE. The roles of carotenoid consumption and bioavailability in cardiovascular health. Antioxidants (Basel, Switzerland). (2021) 10:1978. doi: 10.3390/antiox10121978, PMID: 34943081 PMC8750451

[44] Wang A Han J Jiang Y Zhang D. Association of vitamin a and β-carotene with risk for age-related cataract: a meta-analysis. Nutrition. (2014) 30:1113–21. doi: 10.1016/j.nut.2014.02.025, PMID: 25194611

[45] Cui YH Jing CX Pan HW. Association of blood antioxidants and vitamins with risk of age-related cataract: a meta-analysis of observational studies. Am J Clin Nutr. (2013) 98:778–86. doi: 10.3945/ajcn.112.053835, PMID: 23842458

[46] Giaconi JA Yu F Stone KL Pedula KL Ensrud KE Cauley JA . The association of consumption of fruits/vegetables with decreased risk of glaucoma among older African-American women in the study of osteoporotic fractures. Am J Ophthalmol. (2012) 154:635–44. doi: 10.1016/j.ajo.2012.03.048, PMID: 22818906 PMC3448787

[47] Shalini T Jose SS Prasanthi PS Balakrishna N Viswanath K Reddy GB. Carotenoid status in type 2 diabetes patients with and without retinopathy. Food Funct. (2021) 12:4402–10. doi: 10.1039/d0fo03321a, PMID: 33928954

[48] Jiang H Fan Y Li J Wang J Kong L Wang L . The associations of plasma carotenoids and vitamins with risk of age-related macular degeneration: results from a matched case-control study in China and Meta-analysis. Front Nutr. (2022) 9:745390. doi: 10.3389/fnut.2022.745390, PMID: 35223939 PMC8873933

[49] Vassalle C Maffei S Boni C Zucchelli GC. Gender-related differences in oxidative stress levels among elderly patients with coronary artery disease. Fertil Steril. (2008) 89:608–13. doi: 10.1016/j.fertnstert.2007.03.052, PMID: 17548077

[50] Dreyer L Prescott E Gyntelberg F. Association between atherosclerosis and female lung cancer--a Danish cohort study. Lung Cancer. (2003) 42:247–54. doi: 10.1016/s0169-5002(03)00295-2, PMID: 14644511

[51] Zhou Q Chen X Chen Q Hao L. Independent and combined associations of dietary antioxidant intake with bone mineral density and risk of osteoporosis among elderly population in United States. J Orthop Sci. (2023) 29:1064–72. doi: 10.1016/j.jos.2023.07.014 37537112

[52] Shi WQ Liu J Cao Y Zhu YY Guan K Chen YM. Association of dietary and serum vitamin E with bone mineral density in middle-aged and elderly Chinese adults: a cross-sectional study. Br J Nutr. (2016) 115:113–20. doi: 10.1017/S0007114515004134, PMID: 26507315

[53] Valacchi G Pecorelli A. Role of scavenger receptor B1 (SR-B1) in improving food benefits for human health. Annu Rev Food Sci Technol. (2025) 16:403–32. doi: 10.1146/annurev-food-111523-121935, PMID: 39899837

[54] Ansari KI Kasiri S Hussain I Bobzean SA Perrotti LI Mandal SS. MLL histone methylases regulate expression of HDLR-SR-B1 in presence of estrogen and control plasma cholesterol in vivo. Molecular Endocrinol. (2013) 27:92–105. doi: 10.1210/me.2012-1147, PMID: 23192982 PMC3545218

