# Supplementary material for: Association between serum carotenoid concentrations and risk of major age-related eye diseases among middle-aged and older adults
Source: Front Med (Lausanne). 2025 Dec 1;12:1596799. doi: 10.3389/fmed.2025.1596799 (PMC12702905; doi:10.3389/fmed.2025.1596799)
Supplement: Supplementary file 1 [file Table_1.docx]

**Supplementary Table 1.** Comparison of serum carotenoid concentrations among participants, stratified by sex

|  | Sex | | *P*-value |
| --- | --- | --- | --- |
|  | Male (n = 775) | Female (n = 703) |  |
| α-carotene, μmol/L | 0.08 ± 0.11 | 0.11 ± 0.15 | <0.001 |
| β-cryptoxanthin, μmol/L | 0.36 ± 0.42 | 0.55 ± 0.67 | <0.001 |
| β-cryptoxanthin, μmol/L | 0.18 ± 0.17 | 0.21 ± 0.19 | 0.003 |
| Lycopene, μmol/L | 0.73 ± 0.40 | 0.73 ± 0.37 | 0.993 |
| Lutein/zeaxanthin, μmol/L | 0.31 ± 0.19 | 0.32 ± 0.18 | 0.275 |
| Total carotenoid, μmol/L | 1.66 ± 0.91 | 1.91 ± 1.14 | <0.001 |

**Supplementary Table 2.** Univariate analysis for the association between serum carotenoids concentrations and risk of major age-related eye diseases among elderly population

| Quartiles of serum carotenoids  concentrations | Cataract | | Glaucoma | | Diabetic retinopathy | | Age-related macular degeneration | |
| --- | --- | --- | --- | --- | --- | --- | --- | --- |
|  | Cases/controls | OR (95% CI) | Cases/controls | OR (95% CI) | Cases/controls | OR (95% CI) | Cases/controls | OR (95% CI) |
| α-carotene (μmol/L) |  |  |  |  |  |  |  |  |
| Quartile 1 | 61/284 | 1.00 (Ref) | 32/313 | 1.00 (Ref) | 32/313 | 1.00 (Ref) | 25/320 | 1.00 (Ref) |
| Quartile 2 | 59/324 | 0.85 (0.57-1.25) | 32/351 | 0.89 (0.53-1.49) | 32/351 | 0.89 (0.53-1.49) | 27/356 | 0.97 (0.55-1.71) |
| Quartile 3 | 67/305 | 1.02 (0.70-1.50) | 30/342 | 0.86 (0.51-1.44) | 20/352 | 0.56 (0.31-0.99) | 30/342 | 1.12 (0.65-1.95) |
| Quartile 4 | 43/335 | 0.60 (0.39-0.91) | 19/359 | 0.52 (0.29-0.93) | 10/368 | 0.27 (0.13-0.55) | 28/350 | 1.02 (0.58-1.79) |
| *P* for trend |  | 0.054 |  | 0.036 |  | < 0.001 |  | 0.807 |
| β-carotene (μmol/L) |  |  |  |  |  |  |  |  |
| Quartile 1 | 55/309 | 1.00 (Ref) | 29/335 | 1.00 (Ref) | 37/327 | 1.00 (Ref) | 20/344 | 1.00 (Ref) |
| Quartile 2 | 52/323 | 0.90 (0.60-1.36) | 30/345 | 1.00 (0.59-1.71) | 30/345 | 0.77 (0.46-1.27) | 34/341 | 1.71 (0.97-3.04) |
| Quartile 3 | 60/309 | 1.09 (0.73-1.62) | 23/346 | 0.77 (0.44-1.35) | 19/350 | 0.48 (0.27-0.85) | 18/351 | 0.88 (0.46-1.70) |
| Quartile 4 | 63/307 | 1.15 (0.78-1.71) | 31/339 | 1.06 (0.62-1.79) | 8/362 | 0.20 (0.09-0.43) | 38/332 | 1.97 (1.12-3.45) |
| *P* for trend |  | 0.332 |  | 0.933 |  | < 0.001 |  | 0.100 |
| β-cryptoxanthin (μmol/L) |  |  |  |  |  |  |  |  |
| Quartile 1 | 61/309 | 1.00 (Ref) | 35/335 | 1.00 (Ref) | 32/338 | 1.00 (Ref) | 28/342 | 1.00 (Ref) |
| Quartile 2 | 60/308 | 0.99 (0.67-1.46) | 28/340 | 0.79 (0.47-1.32) | 32/336 | 1.01 (0.60-1.68) | 22/346 | 0.78 (0.44-1.38) |
| Quartile 3 | 63/307 | 1.04 (0.71-1.53) | 19/351 | 0.52 (0.29-0.92) | 17/353 | 0.51 (0.28-0.93) | 39/331 | 1.44 (0.87-2.39) |
| Quartile 4 | 46/324 | 0.72 (0.48-1.09) | 31/339 | 0.88 (0.53-1.45) | 13/357 | 0.38 (0.20-0.75) | 21/349 | 0.73 (0.41-1.32) |
| *P* for trend |  | 0.175 |  | 0.355 |  | < 0.001 |  | 0.854 |
| Lycopene (μmol/L) |  |  |  |  |  |  |  |  |
| Quartile 1 | 83/286 | 1.00 (Ref) | 41/328 | 1.00 (Ref) | 29/340 | 1.00 (Ref) | 35/334 | 1.00 (Ref) |
| Quartile 2 | 62/307 | 0.70 (0.48-1.00) | 26/343 | 0.61 (0.36-1.01) | 22/347 | 0.74 (0.42-1.32) | 35/334 | 1.00 (0.61-1.64) |
| Quartile 3 | 50/320 | 0.54 (0.37-0.79) | 25/345 | 0.58 (0.34-0.97) | 25/345 | 0.85 (0.49-1.48) | 29/341 | 0.81 (0.49-1.36) |
| Quartile 4 | 35/335 | 0.36 (0.24-0.55) | 21/349 | 0.48 (0.28-0.83) | 18/352 | 0.60 (0.33-1.10) | 11/359 | 0.29 (0.15-0.59) |
| *P* for trend |  | < 0.001 |  | 0.008 |  | 0.150 |  | < 0.001 |
| Lutein/zeaxanthin (μmol/L) |  |  |  |  |  |  |  |  |
| Quartile 1 | 71/299 | 1.00 (Ref) | 25/345 | 1.00 (Ref) | 32/338 | 1.00 (Ref) | 25/345 | 1.00 (Ref) |
| Quartile 2 | 65/298 | 0.90 (0.62-1.31) | 26/337 | 1.09 (0.62-1.92) | 21/342 | 0.64 (0.36-1.13) | 32/331 | 0.77 (0.45-1.33) |
| Quartile 3 | 59/313 | 0.81 (0.55-1.18) | 37/335 | 1.50 (0.88-2.56) | 20/352 | 0.61 (0.34-1.09) | 28/344 | 0.77 (0.44-1.32) |
| Quartile 4 | 35/338 | 0.44 (0.28-0.67) | 25/348 | 0.99 (0.56-1.76) | 21/352 | 0.63 (0.36-1.11) | 25/348 | 0.86 (0.51-1.47) |
| *P* for trend |  | < 0.001 |  | 0.714 |  | 0.102 |  | 0.839 |
| Total carotenoid (μmol/L) |  |  |  |  |  |  |  |  |
| Quartile 1 | 76/294 | 1.00 (Ref) | 35/335 | 1.00 (Ref) | 33/337 | 1.00 (Ref) | 32/338 | 1.00 (Ref) |
| Quartile 2 | 63/305 | 0.80 (0.55-1.16) | 31/337 | 0.88 (0.53-1.46) | 29/339 | 0.87 (0.52-1.47) | 25/343 | 0.77 (0.45-1.33) |
| Quartile 3 | 48/322 | 0.58 (0.39-0.86) | 22/348 | 0.61 (0.35-1.05) | 19/351 | 0.55 (0.31-0.99) | 25/345 | 0.77 (0.44-1.32) |
| Quartile 4 | 43/327 | 0.51 (0.34-0.76) | 25/345 | 0.69 (0.41-1.18) | 13/357 | 0.37 (0.19-0.72) | 28/342 | 0.86 (0.51-1.47) |
| *P* for trend |  | < 0.001 |  | 0.088 |  | 0.001 |  | 0.591 |

*P* for trend were obtained by including quartile number as a continuous variable in the regression model.

**Supplementary Table 3.** Sensitivity analysis of the association between serum carotenoids and risk of age-related eye diseases, with additional adjustment for dietary carotenoid intake

| Quartiles of serum carotenoids  concentrations | Cataract | Glaucoma | Diabetic retinopathy | Age-related macular degeneration | Any ocular disease |
| --- | --- | --- | --- | --- | --- |
|  | OR (95% CI) | OR (95% CI) | OR (95% CI) | OR (95% CI) | OR (95% CI) |
| α-carotene (μmol/L) |  |  |  |  |  |
| Per 1-SD increase | 0.87 (0.65, 1.17) | 0.75 (0.58, 0.96) | 0.62 (0.36, 1.05) | 1.08 (0.86, 1.35) | 0.82 (0.68, 0.98) |
| Quartile 1 | 1.00 (Ref) | 1.00 (Ref) | 1.00 (Ref) | 1.00 (Ref) | 1.00 (Ref) |
| Quartile 2 | 0.93 (0.54, 1.62) | 0.62 (0.38, 1.01) | 1.10 (0.57, 2.11) | 0.74 (0.40, 1.37) | 0.96 (0.67, 1.38) |
| Quartile 3 | 0.77 (0.43, 1.38) | 0.61 (0.37, 0.99) | 0.80 (0.37, 1.70) | 0.70 (0.37, 1.31) | 0.72 (0.49, 1.06) |
| Quartile 4 | 0.58 (0.30, 1.13) | 0.37 (0.21, 0.65) | 0.37 (0.15, 0.90) | 0.70 (0.36, 1.35) | 0.47 (0.31, 0.71) |
| *P* for trend | 0.091 | 0.001 | 0.032 | 0.314 | <0.001 |
| β-carotene (μmol/L) |  |  |  |  |  |
| Per 1-SD increase | 0.91 (0.71, 1.17) | 0.96 (0.81, 1.13) | 0.32 (0.16, 0.67) | 1.20 (1.02, 1.40) | 0.96 (0.84, 1.10) |
| Quartile 1 | 1.00 (Ref) | 1.00 (Ref) | 1.00 (Ref) | 1.00 (Ref) | 1.00 (Ref) |
| Quartile 2 | 0.82 (0.46, 1.45) | 0.63 (0.38, 1.05) | 1.00 (0.53, 1.89) | 1.51 (0.81, 2.81) | 0.81 (0.56, 1.17) |
| Quartile 3 | 0.56 (0.30, 1.06) | 0.66 (0.40, 1.09) | 0.71 (0.34, 1.50) | 0.60 (0.29, 1.22) | 0.55 (0.37, 0.81) |
| Quartile 4 | 0.78 (0.42, 1.45) | 0.58 (0.34, 0.99) | 0.24 (0.09, 0.64) | 1.40 (0.72, 2.72) | 0.55 (0.36, 0.82) |
| *P* for trend | 0.319 | 0.077 | 0.007 | 0.855 | 0.001 |
| β-cryptoxanthin (μmol/L) |  |  |  |  |  |
| Per 1-SD increase | 1.04 (0.83, 1.30) | 0.89 (0.70, 1.11) | 0.50 (0.32, 0.77) | 0.84 (0.62, 1.13) | 0.90 (0.77, 1.06) |
| Quartile 1 | 1.00 (Ref) | 1.00 (Ref) | 1.00 (Ref) | 1.00 (Ref) | 1.00 (Ref) |
| Quartile 2 | 0.78 (0.45, 1.35) | 1.07 (0.67, 1.72) | 0.87 (0.45, 1.68) | 0.81 (0.44, 1.49) | 0.95 (0.66, 1.37) |
| Quartile 3 | 0.51 (0.27, 0.94) | 1.16 (0.72, 1.89) | 0.52 (0.24, 1.12) | 1.40 (0.79, 2.48) | 1.03 (0.71, 1.50) |
| Quartile 4 | 0.91 (0.51, 1.64) | 0.69 (0.41, 1.17) | 0.22 (0.09, 0.54) | 0.69 (0.36, 1.34) | 0.67 (0.45, 1.00) |
| *P* for trend | 0.482 | 0.262 | <0.001 | 0.673 | 0.089 |
| Lycopene (μmol/L) |  |  |  |  |  |
| Per 1-SD increase | 0.97 (0.77, 1.21) | 1.02 (0.83, 1.25) | 0.92 (0.69, 1.22) | 0.75 (0.57, 0.98) | 0.91 (0.79, 1.06) |
| Quartile 1 | 1.00 (Ref) | 1.00 (Ref) | 1.00 (Ref) | 1.00 (Ref) | 1.00 (Ref) |
| Quartile 2 | 0.61 (0.36, 1.06) | 0.86 (0.56, 1.34) | 0.82 (0.40, 1.65) | 1.12 (0.66, 1.90) | 0.81 (0.57, 1.14) |
| Quartile 3 | 0.70 (0.39, 1.22) | 1.01 (0.63, 1.62) | 0.95 (0.46, 1.95) | 0.98 (0.55, 1.76) | 1.00 (0.70, 1.44) |
| Quartile 4 | 0.69 (0.38, 1.26) | 0.89 (0.53, 1.49) | 0.82 (0.38, 1.76) | 0.39 (0.18, 0.83) | 0.63 (0.43, 0.94) |
| *P* for trend | 0.228 | 0.785 | 0.703 | 0.033 | 0.086 |
| Lutein/zeaxanthin (μmol/L) |  |  |  |  |  |
| Per 1-SD increase | 0.99 (0.79, 1.23) | 0.81 (0.67, 0.99) | 0.99 (0.77, 1.27) | 1.32 (1.10, 1.58) | 1.02 (0.89, 1.16) |
| Quartile 1 | 1.00 (Ref) | 1.00 (Ref) | 1.00 (Ref) | 1.00 (Ref) | 1.00 (Ref) |
| Quartile 2 | 0.97 (0.53, 1.77) | 0.95 (0.60, 1.52) | 0.55 (0.26, 1.14) | 1.36 (0.75, 2.47) | 1.00 (0.69, 1.45) |
| Quartile 3 | 1.36 (0.76, 2.43) | 0.83 (0.51, 1.34) | 0.64 (0.30, 1.36) | 1.23 (0.66, 2.27) | 1.08 (0.74, 1.57) |
| Quartile 4 | 0.97 (0.52, 1.83) | 0.46 (0.27, 0.79) | 0.51 (0.24, 1.10) | 1.19 (0.62, 2.27) | 0.71 (0.48, 1.05) |
| *P* for trend | 0.768 | 0.006 | 0.134 | 0.711 | 0.123 |
| Total carotenoid (μmol/L) |  |  |  |  |  |
| Per 1-SD increase | 0.93 (0.73, 1.18) | 0.90 (0.74, 1.09) | 0.63 (0.42, 0.93) | 1.14 (0.92, 1.40) | 0.92 (0.79, 1.06) |
| Quartile 1 | 1.00 (Ref) | 1.00 (Ref) | 1.00 (Ref) | 1.00 (Ref) | 1.00 (Ref) |
| Quartile 2 | 0.82 (0.47, 1.42) | 1.01 (0.64, 1.59) | 0.85 (0.43, 1.70) | 0.83 (0.46, 1.50) | 1.05 (0.73, 1.50) |
| Quartile 3 | 0.58 (0.32, 1.07) | 0.60 (0.37, 0.98) | 0.84 (0.40, 1.77) | 0.73 (0.40, 1.36) | 0.71 (0.48, 1.03) |
| Quartile 4 | 0.80 (0.43, 1.47) | 0.59 (0.35, 1.00) | 0.41 (0.18, 0.95) | 0.94 (0.50, 1.76) | 0.63 (0.42, 0.94) |
| *P* for trend | 0.281 | 0.014 | 0.055 | 0.755 | 0.007 |

*P* for trend were obtained by including quartile number as a continuous variable in the regression model. Models were adjusted for age (continuous), sex (male or female), race/ethnicity (non-Hispanic white, black, Mexican-American, other Hispanic, or other race/ethnicity), education level (less than high school, high school or equivalent, or college or above), family income-to-poverty ratio (<1.3, 1.3 to ≤3.5, >3.5, or missing), body mass index (<18.5, 18.5 to <25, 25 to <30, ≥30 kg/m^2^, or missing), drinking status (nondrinker or drinker), smoking status (never smoker, former smoker, current smoker, or missing), HbA_1c_ (<6.5%, ≥6.5%, or missing), hypertension (yes, no, or missing), hypercholesterolemia (yes, no, or missing), cancer (yes, no, or missing), and dietary carotenoid intake (quartiles or missing)

**Supplementary Table 4.** Association between serum carotenoids concentrations and risk of major age-related eye diseases among middle-aged and older adults after excluding participants with missing data on covariates

| Quartiles of serum carotenoids  concentrations | Cataract | | Glaucoma | | Diabetic retinopathy | | Age-related macular degeneration | |
| --- | --- | --- | --- | --- | --- | --- | --- | --- |
|  | Cases/controls | OR (95% CI) | Cases/controls | OR (95% CI) | Cases/controls | OR (95% CI) | Cases/controls | OR (95% CI) |
| α-carotene (μmol/L) |  |  |  |  |  |  |  |  |
| Per 1-SD increase |  | 0.75 (0.58, 0.97) |  | 0.89 (0.67, 1.20) |  | 0.65 (0.38, 1.12) |  | 1.11 (0.89, 1.38) |
| Quartile 1 | 46/213 | 1.00 (Ref) | 27/232 | 1.00 (Ref) | 27/232 | 1.00 (Ref) | 22/237 | 1.00 (Ref) |
| Quartile 2 | 49/254 | 0.58 (0.34, 0.99) | 29/274 | 0.95 (0.53, 1.72) | 27/276 | 1.14 (0.56, 2.30) | 21/282 | 0.57 (0.29, 1.12) |
| Quartile 3 | 55/236 | 0.62 (0.36, 1.07) | 25/266 | 0.69 (0.37, 1.30) | 15/276 | 0.89 (0.39, 2.02) | 23/268 | 0.56 (0.28, 1.12) |
| Quartile 4 | 30/274 | 0.31 (0.17, 0.58) | 18/286 | 0.61 (0.30, 1.22) | 8/296 | 0.34 (0.13, 0.91) | 23/281 | 0.61 (0.30, 1.24) |
| *P* for trend |  | <0.001 |  | 0.098 |  | 0.005 |  | 0.150 |
| β-carotene (μmol/L) |  |  |  |  |  |  |  |  |
| Per 1-SD increase |  | 0.95 (0.77, 1.16) |  | 0.96 (0.74, 1.24) |  | 0.33 (0.15, 0.71) |  | 1.15 (0.95, 1.38) |
| Quartile 1 | 41/233 | 1.00 (Ref) | 25/249 | 1.00 (Ref) | 31/243 | 1.00 (Ref) | 18/256 | 1.00 (Ref) |
| Quartile 2 | 44/248 | 0.64 (0.37, 1.13) | 25/267 | 0.77 (0.41, 1.42) | 24/268 | 1.00 (0.50, 1.99) | 25/267 | 1.11 (0.56, 2.18) |
| Quartile 3 | 47/244 | 0.60 (0.34, 1.05) | 19/272 | 0.51 (0.26, 1.00) | 15/276 | 0.71 (0.32, 1.57) | 17/274 | 0.57 (0.27, 1.21) |
| Quartile 4 | 48/252 | 0.53 (0.30, 0.96) | 30/270 | 0.86 (0.45, 1.63) | 7/293 | 0.27 (0.09, 0.75) | 29/271 | 1.05 (0.51, 2.13) |
| *P* for trend |  | 0.049 |  | 0.507 |  | 0.008 |  | 0.312 |
| β-cryptoxanthin (μmol/L) |  |  |  |  |  |  |  |  |
| Per 1-SD increase |  | 0.78 (0.60, 1.02) |  | 1.02 (0.79, 1.32) |  | 0.50 (0.30, 0.83) |  | 0.85 (0.61, 1.17) |
| Quartile 1 | 46/246 | 1.00 (Ref) | 28/264 | 1.00 (Ref) | 27/265 | 1.00 (Ref) | 23/269 | 1.00 (Ref) |
| Quartile 2 | 45/241 | 0.98 (0.58, 1.66) | 25/261 | 0.83 (0.46, 1.51) | 30/256 | 1.07 (0.54, 2.12) | 18/268 | 0.77 (0.40, 1.51) |
| Quartile 3 | 52/244 | 1.15 (0.68, 1.95) | 18/278 | 0.57 (0.30, 1.10) | 11/285 | 0.37 (0.15, 0.89) | 31/265 | 1.25 (0.67, 2.31) |
| Quartile 4 | 37/246 | 0.75 (0.42, 1.33) | 28/255 | 0.97 (0.53, 1.79) | 9/274 | 0.24 (0.09, 0.65) | 17/266 | 0.69 (0.34, 1.41) |
| *P* for trend |  | 0.490 |  | 0.684 |  | 0.621 |  | 0.032 |
| Lycopene (μmol/L) |  |  |  |  |  |  |  |  |
| Per 1-SD increase |  | 1.06 (0.85, 1.31) |  | 0.98 (0.77, 1.24) |  | 0.93 (0.69, 1.25) |  | 0.73 (0.55, 0.97) |
| Quartile 1 | 58/214 | 1.00 (Ref) | 34/238 | 1.00 (Ref) | 23/249 | 1.00 (Ref) | 27/245 | 1.00 (Ref) |
| Quartile 2 | 48/233 | 0.94 (0.57, 1.56) | 21/260 | 0.62 (0.34, 1.13) | 18/263 | 1.03 (0.48, 2.21) | 30/251 | 1.16 (0.65, 2.07) |
| Quartile 3 | 44/257 | 1.16 (0.69, 1.93) | 25/276 | 0.80 (0.45, 1.43) | 22/279 | 1.09 (0.52, 2.31) | 22/279 | 0.83 (0.44, 1.57) |
| Quartile 4 | 30/273 | 1.05 (0.59, 1.85) | 19/284 | 0.72 (0.38, 1.35) | 14/289 | 0.81 (0.35, 1.84) | 10/293 | 0.41 (0.18, 0.91) |
| *P* for trend |  | 0.692 |  | 0.386 |  | 0.982 |  | 0.004 |
| Lutein/zeaxanthin (μmol/L) |  |  |  |  |  |  |  |  |
| Per 1-SD increase |  | 0.79 (0.63, 0.98) |  | 0.94 (0.75, 1.19) |  | 1.00 (0.76, 1.31) |  | 1.34 (1.10, 1.64) |
| Quartile 1 | 54/239 | 1.00 (Ref) | 24/269 | 1.00 (Ref) | 27/266 | 1.00 (Ref) | 18/275 | 1.00 (Ref) |
| Quartile 2 | 50/228 | 0.85 (0.50, 1.43) | 21/257 | 0.78 (0.41, 1.48) | 19/259 | 0.70 (0.33, 1.52) | 27/251 | 1.46 (0.75, 2.84) |
| Quartile 3 | 49/239 | 0.80 (0.47, 1.36) | 32/256 | 1.18 (0.65, 2.17) | 16/272 | 0.68 (0.30, 1.53) | 23/265 | 1.21 (0.60, 2.42) |
| Quartile 4 | 27/271 | 0.41 (0.23, 0.75) | 22/276 | 0.79 (0.41, 1.51) | 15/283 | 0.55 (0.24, 1.26) | 21/277 | 1.19 (0.58, 2.41) |
| *P* for trend |  | 0.006 |  | 0.799 |  | 0.046 |  | 0.520 |
| Total carotenoid (μmol/L) |  |  |  |  |  |  |  |  |
| Per 1-SD increase |  | 0.88 (0.71, 1.10) |  | 0.95 (0.74, 1.22) |  | 0.66 (0.44, 0.99) |  | 1.08 (0.85, 1.36) |
| Quartile 1 | 58/224 | 1.00 (Ref) | 29/253 | 1.00 (Ref) | 29/253 | 1.00 (Ref) | 26/256 | 1.00 (Ref) |
| Quartile 2 | 50/235 | 0.96 (0.58, 1.58) | 27/258 | 0.88 (0.49, 1.58) | 23/262 | 0.86 (0.42, 1.78) | 21/264 | 0.79 (0.41, 1.49) |
| Quartile 3 | 36/255 | 0.56 (0.32, 0.96) | 19/272 | 0.59 (0.31, 1.12) | 16/275 | 0.89 (0.41, 1.94) | 19/272 | 0.62 (0.32, 1.21) |
| Quartile 4 | 36/263 | 0.60 (0.34, 1.06) | 24/275 | 0.86 (0.46, 1.61) | 9/290 | 0.37 (0.15, 0.95) | 23/276 | 0.83 (0.42, 1.62) |
| *P* for trend |  | 0.023 |  | 0.394 |  | 0.068 |  | 0.457 |

*P* for trend were obtained by including quartile number as a continuous variable in the regression model. Models were adjusted for age (continuous), sex (male or female), race/ethnicity (non-Hispanic white, black, Mexican-American, other Hispanic, or other race/ethnicity), education level (less than high school, high school or equivalent, or college or above), family income-to-poverty ratio (<1.3, 1.3 to ≤3.5, >3.5, or missing), body mass index (<18.5, 18.5 to <25, 25 to <30, ≥30 kg/m^2^, or missing), drinking status (nondrinker or drinker), smoking status (never smoker, former smoker, current smoker, or missing), HbA_1c_ (<6.5%, ≥6.5%, or missing), hypertension (yes, no, or missing), hypercholesterolemia (yes, no, or missing), and cancer (yes, no, or missing).

**Supplementary Table 5.** Association between serum carotenoids concentrations and risk of any ocular disease among middle-aged and older adults after excluding participants with missing data on covariates

|  | Quartiles of serum carotenoids concentrations (μmol/L) | | | | Per 1-SD increase | *P* for trend |
| --- | --- | --- | --- | --- | --- | --- |
|  | Quartile 1 | Quartile 2 | Quartile 3 | Quartile 4 |  |  |
|  | OR (95% CI) | OR (95% CI) | OR (95% CI) | OR (95% CI) |  |  |
| α-carotene |  |  |  |  |  |  |
| Cases/controls | 88/171 | 106/197 | 89/202 | 67/237 |  |  |
| Multivariate model | 1.00 (Ref) | 0.91 (0.61, 1.37) | 0.65 (0.42, 1.01) | 0.46 (0.29, 0.73) | 0.86 (0.72, 1.03) | < 0.001 |
| β-carotene |  |  |  |  |  |  |
| Cases/controls | 93/181 | 92/200 | 80/211 | 85/215 |  |  |
| Multivariate model | 1.00 (Ref) | 0.75 (0.50, 1.14) | 0.49 (0.32, 0.76) | 0.53 (0.34, 0.82) | 0.99 (0.85, 1.15) | 0.002 |
| β-cryptoxanthin |  |  |  |  |  |  |
| Cases/controls | 92/200 | 92/194 | 92/204 | 74/209 |  |  |
| Multivariate model | 1.00 (Ref) | 1.00 (0.67, 1.49) | 0.97 (0.65, 1.46) | 0.71 (0.46, 1.11) | 0.84 (0.70, 1.01) | 0.153 |
| Lycopene |  |  |  |  |  |  |
| Cases/controls | 105/167 | 90/191 | 96/205 | 59/244 |  |  |
| Multivariate model | 1.00 (Ref) | 0.94 (0.63, 1.40) | 1.15 (0.77, 1.70) | 0.71 (0.46, 1.09) | 0.93 (0.80, 1.09) | 0.287 |
| Lutein/zeaxanthin |  |  |  |  |  |  |
| Cases/controls | 92/201 | 92/186 | 96/192 | 70/228 |  |  |
| Multivariate model | 1.00 (Ref) | 0.95 (0.63, 1.43) | 0.98 (0.65, 1.48) | 0.62 (0.40, 0.96) | 1.00 (0.87, 1.16) | 0.044 |
| Total carotenoid |  |  |  |  |  |  |
| Cases/controls | 103/179 | 99/186 | 75/216 | 73/226 |  |  |
| Multivariate model | 1.00 (Ref) | 1.05 (0.70, 1.56) | 0.65 (0.43, 0.98) | 0.65 (0.42, 1.01) | 0.93 (0.79, 1.09) | 0.053 |

*P* for trend were obtained by including quartile number as a continuous variable in the regression model. Models were adjusted for age (continuous), sex (male or female), race/ethnicity (non-Hispanic white, black, Mexican-American, other Hispanic, or other race/ethnicity), education level (less than high school, high school or equivalent, college or above, or missing), family income-to-poverty ratio (<1.3, 1.3 to ≤3.5, >3.5, or missing), body mass index (<18.5, 18.5 to <25, 25 to <30, ≥30 kg/m2, or missing), drinking status (nondrinker or drinker), smoking status (never smoker, former smoker, current smoker, or missing), HbA1c (<6.5%, ≥6.5%, or missing), hypertension (yes, no, or missing), hypercholesterolemia (yes, no, or missing), and cancer (yes, no, or missing).

**Supplementary Table 6.** Association between serum carotenoids concentrations and risk of cataract among middle-aged and older adults by sex

| Quartiles of serum carotenoids concentrations | Male | | Female | | *P* for interaction |
| --- | --- | --- | --- | --- | --- |
|  | Cases/controls | OR (95% CI) | Cases/controls | OR (95% CI) |  |
| α-carotene |  |  |  |  | 0.006 |
| Per 1-SD increase |  | 1.09 (0.77, 1.54) |  | 0.52 (0.34, 0.78) |  |
| Quartile 1 | 31/184 | 1.00 (Ref) | 30/100 | 1.00 (Ref) |  |
| Quartile 2 | 29/182 | 0.87 (0.45, 1.68) | 30/142 | 0.35 (0.16, 0.77) |  |
| Quartile 3 | 26/164 | 0.60 (0.30, 1.21) | 41/141 | 0.51 (0.23, 1.11) |  |
| Quartile 4 | 21/138 | 0.74 (0.35, 1.55) | 22/197 | 0.15 (0.06, 0.36) |  |
| *P* for trend |  | 0.259 |  | <0.001 |  |
| β-carotene |  |  |  |  | 0.709 |
| Per 1-SD increase |  | 0.98 (0.72, 1.34) |  | 0.91 (0.73, 1.13) |  |
| Quartile 1 | 34/207 | 1.00 (Ref) | 21/102 | 1.00 (Ref) |  |
| Quartile 2 | 24/179 | 0.66 (0.34, 1.27) | 28/144 | 0.48 (0.21, 1.11) |  |
| Quartile 3 | 28/165 | 0.77 (0.40, 1.47) | 32/144 | 0.43 (0.18, 1.00) |  |
| Quartile 4 | 21/117 | 0.64 (0.31, 1.34) | 42/190 | 0.42 (0.18, 0.97) |  |
| *P* for trend |  | 0.291 |  | 0.079 |  |
| β-cryptoxanthin |  |  |  |  | <0.001 |
| Per 1-SD increase |  | 1.15 (0.93, 1.43) |  | 0.52 (0.34, 0.80) |  |
| Quartile 1 | 31/186 | 1.00 (Ref) | 30/123 | 1.00 (Ref) |  |
| Quartile 2 | 25/168 | 0.92 (0.48, 1.77) | 35/140 | 1.29 (0.63, 2.68) |  |
| Quartile 3 | 29/162 | 0.95 (0.49, 1.84) | 34/145 | 1.29 (0.60, 2.79) |  |
| Quartile 4 | 22/152 | 0.95 (0.46, 1.93) | 24/172 | 0.52 (0.23, 1.19) |  |
| *P* for trend |  | 0.894 |  | 0.136 |  |
| Lycopene |  |  |  |  | 0.489 |
| Per 1-SD increase |  | 1.07 (0.82, 1.39) |  | 0.92 (0.67, 1.27) |  |
| Quartile 1 | 38/161 | 1.00 (Ref) | 45/125 | 1.00 (Ref) |  |
| Quartile 2 | 30/153 | 0.93 (0.50, 1.72) | 32/154 | 0.77 (0.40, 1.50) |  |
| Quartile 3 | 20/176 | 0.77 (0.39, 1.52) | 30/144 | 1.21 (0.60, 2.45) |  |
| Quartile 4 | 19/178 | 1.01 (0.51, 2.02) | 16/157 | 0.71 (0.32, 1.61) |  |
| *P* for trend |  | 0.832 |  | 0.746 |  |
| Lutein/zeaxanthin |  |  |  |  | 0.046 |
| Per 1-SD increase |  | 0.92 (0.71, 1.17) |  | 0.60 (0.43, 0.84) |  |
| Quartile 1 | 32/162 | 1.00 (Ref) | 39/137 | 1.00 (Ref) |  |
| Quartile 2 | 30/175 | 0.94 (0.49, 1.80) | 35/128 | 0.68 (0.32, 1.43) |  |
| Quartile 3 | 29/164 | 1.11 (0.57, 2.14) | 30/144 | 0.53 (0.24, 1.15) |  |
| Quartile 4 | 16/167 | 0.52 (0.25, 1.10) | 19/171 | 0.26 (0.11, 0.61) |  |
| *P* for trend |  | 0.178 |  | 0.002 |  |
| Total carotenoid |  |  |  |  | 0.086 |
| Per 1-SD increase |  | 1.04 (0.78, 1.38) |  | 0.73 (0.55, 0.97) |  |
| Quartile 1 | 42/172 | 1.00 (Ref) | 34/117 | 1.00 (Ref) |  |
| Quartile 2 | 25/168 | 0.71 (0.38, 1.34) | 38/137 | 1.26 (0.62, 2.57) |  |
| Quartile 3 | 18/180 | 0.48 (0.24, 0.95) | 30/142 | 0.62 (0.29, 1.32) |  |
| Quartile 4 | 22/143 | 0.85 (0.43, 1.71) | 21/184 | 0.35 (0.15, 0.80) |  |
| *P* for trend |  | 0.292 |  | 0.005 |  |

*P* for trend were obtained by including quartile number as a continuous variable in the regression model. *P* for interaction was calculated using the likelihood ratio test. Models were adjusted for age (continuous), race/ethnicity (non-Hispanic white, black, Mexican-American, other Hispanic, or other race/ethnicity), education level (less than high school, high school or equivalent, college or above, or missing), family income-to-poverty ratio (<1.3, 1.3 to ≤3.5, >3.5, or missing), body mass index (<18.5, 18.5 to <25, 25 to <30, ≥30 kg/m^2^, or missing), drinking status (nondrinker or drinker), smoking status (never smoker, former smoker, current smoker, or missing), HbA_1c_ (<6.5%, ≥6.5%, or missing), hypertension (yes, no, or missing), hypercholesterolemia (yes, no, or missing), and cancer (yes, no, or missing).

**Supplementary Table 7.** Association between serum carotenoids concentrations and risk of glaucoma among middle-aged and older adults by sex

| Quartiles of serum carotenoids concentrations | Male | | Female | | *P* for interaction |
| --- | --- | --- | --- | --- | --- |
|  | Cases/controls | OR (95% CI) | Cases/controls | OR (95% CI) |  |
| α-carotene |  |  |  |  | 0.283 |
| Per 1-SD increase |  | 1.00 (0.70, 1.44) |  | 0.73 (0.46, 1.16) |  |
| Quartile 1 | 16/199 | 1.00 (Ref) | 16/114 | 1.00 (Ref) |  |
| Quartile 2 | 20/191 | 1.45 (0.69, 3.06) | 12/160 | 0.51 (0.22, 1.22) |  |
| Quartile 3 | 12/178 | 0.82 (0.35, 1.91) | 18/164 | 0.56 (0.24, 1.29) |  |
| Quartile 4 | 11/148 | 1.12 (0.46, 2.73) | 8/211 | 0.24 (0.09, 0.67) |  |
| *P* for trend |  | 0.822 |  | 0.012 |  |
| β-carotene |  |  |  |  | 0.483 |
| Per 1-SD increase |  | 1.02 (0.69, 1.49) |  | 0.84 (0.59, 1.20) |  |
| Quartile 1 | 19/222 | 1.00 (Ref) | 10/113 | 1.00 (Ref) |  |
| Quartile 2 | 17/186 | 0.88 (0.43, 1.82) | 13/159 | 0.67 (0.26, 1.71) |  |
| Quartile 3 | 10/183 | 0.46 (0.20, 1.09) | 13/163 | 0.57 (0.22, 1.50) |  |
| Quartile 4 | 13/125 | 0.96 (0.42, 2.20) | 18/214 | 0.62 (0.24, 1.59) |  |
| *P* for trend |  | 0.496 |  | 0.383 |  |
| β-cryptoxanthin |  |  |  |  | 0.892 |
| Per 1-SD increase |  | 1.00 (0.71, 1.41) |  | 1.04 (0.75, 1.42) |  |
| Quartile 1 | 20/197 | 1.00 (Ref) | 15/138 | 1.00 (Ref) |  |
| Quartile 2 | 13/180 | 0.68 (0.31, 1.47) | 15/160 | 0.70 (0.31, 1.58) |  |
| Quartile 3 | 12/179 | 0.64 (0.29, 1.42) | 7/172 | 0.32 (0.12, 0.87) |  |
| Quartile 4 | 14/160 | 0.88 (0.40, 1.93) | 17/179 | 0.80 (0.33, 1.91) |  |
| *P* for trend |  | 0.647 |  | 0.411 |  |
| Lycopene |  |  |  |  | 0.229 |
| Per 1-SD increase |  | 1.12 (0.83, 1.50) |  | 0.84 (0.59, 1.21) |  |
| Quartile 1 | 18/181 | 1.00 (Ref) | 23/147 | 1.00 (Ref) |  |
| Quartile 2 | 11/172 | 0.68 (0.30, 1.54) | 15/171 | 0.58 (0.28, 1.23) |  |
| Quartile 3 | 19/177 | 1.30 (0.62, 2.73) | 6/168 | 0.29 (0.11, 0.77) |  |
| Quartile 4 | 11/186 | 0.93 (0.40, 2.15) | 10/163 | 0.59 (0.25, 1.42) |  |
| *P* for trend |  | 0.770 |  | 0.076 |  |
| Lutein/zeaxanthin |  |  |  |  | 0.877 |
| Per 1-SD increase |  | 0.97 (0.72, 1.30) |  | 0.93 (0.67, 1.31) |  |
| Quartile 1 | 13/181 | 1.00 (Ref) | 12/164 | 1.00 (Ref) |  |
| Quartile 2 | 12/193 | 0.86 (0.36, 2.03) | 15/148 | 1.04 (0.44, 2.50) |  |
| Quartile 3 | 21/172 | 1.58 (0.72, 3.44) | 15/159 | 1.01 (0.41, 2.46) |  |
| Quartile 4 | 13/170 | 1.00 (0.43, 2.36) | 12/178 | 0.74 (0.29, 1.89) |  |
| *P* for trend |  | 0.606 |  | 0.518 |  |
| Total carotenoid |  |  |  |  | 0.296 |
| Per 1-SD increase |  | 1.05 (0.76, 1.47) |  | 0.81 (0.56, 1.17) |  |
| Quartile 1 | 19/200 | 1.00 (Ref) | 16/135 | 1.00 (Ref) |  |
| Quartile 2 | 14/179 | 0.75 (0.34, 1.61) | 17/158 | 0.89 (0.40, 1.95) |  |
| Quartile 3 | 13/185 | 0.75 (0.34, 1.66) | 9/163 | 0.40 (0.16, 1.02) |  |
| Quartile 4 | 13/152 | 1.16 (0.51, 2.63) | 12/193 | 0.55 (0.22, 1.34) |  |
| *P* for trend |  | 0.857 |  | 0.077 |  |

*P* for trend were obtained by including quartile number as a continuous variable in the regression model. *P* for interaction was calculated using the likelihood ratio test. Models were adjusted for age (continuous), race/ethnicity (non-Hispanic white, black, Mexican-American, other Hispanic, or other race/ethnicity), education level (less than high school, high school or equivalent, college or above, or missing), family income-to-poverty ratio (<1.3, 1.3 to ≤3.5, >3.5, or missing), body mass index (<18.5, 18.5 to <25, 25 to <30, ≥30 kg/m^2^, or missing), drinking status (nondrinker or drinker), smoking status (never smoker, former smoker, current smoker, or missing), HbA_1c_ (<6.5%, ≥6.5%, or missing), hypertension (yes, no, or missing), hypercholesterolemia (yes, no, or missing), and cancer (yes, no, or missing).

**Supplementary Table 8.** Association between serum carotenoids concentrations and risk of diabetic retinopathy among middle-aged and older adults by sex

| Quartiles of serum carotenoids concentrations | Male | | Female | | *P* for interaction |
| --- | --- | --- | --- | --- | --- |
|  | Cases/controls | OR (95% CI) | Cases/controls | OR (95% CI) |  |
| α-carotene |  |  |  |  | 0.334 |
| Per 1-SD increase |  | 0.93 (0.48, 1.80) |  | 0.56 (0.27, 1.17) |  |
| Quartile 1 | 16/199 | 1.00 (Ref) | 16/114 | 1.00 (Ref) |  |
| Quartile 2 | 21/190 | 2.00 (0.83, 4.81) | 11/161 | 0.59 (0.20, 1.75) |  |
| Quartile 3 | 10/180 | 1.46 (0.52, 4.10) | 10/172 | 0.56 (0.16, 1.92) |  |
| Quartile 4 | 4/155 | 0.84 (0.22, 3.19) | 6/213 | 0.22 (0.06, 0.82) |  |
| *P* for trend |  | 0.989 |  | 0.030 |  |
| β-carotene |  |  |  |  | 0.167 |
| Per 1-SD increase |  | 0.59 (0.24, 1.41) |  | 0.21 (0.06, 0.69) |  |
| Quartile 1 | 23/218 | 1.00 (Ref) | 14/109 | 1.00 (Ref) |  |
| Quartile 2 | 13/190 | 0.80 (0.32, 1.96) | 17/155 | 1.54 (0.55, 4.32) |  |
| Quartile 3 | 12/181 | 1.11 (0.43, 2.84) | 7/169 | 0.59 (0.17, 2.08) |  |
| Quartile 4 | 3/135 | 0.32 (0.07, 1.39) | 5/227 | 0.24 (0.06, 0.95) |  |
| *P* for trend |  | 0.309 |  | 0.021 |  |
| β-cryptoxanthin |  |  |  |  | 0.764 |
| Per 1-SD increase |  | 0.50 (0.26, 0.96) |  | 0.58 (0.32, 1.04) |  |
| Quartile 1 | 20/197 | 1.00 (Ref) | 12/141 | 1.00 (Ref) |  |
| Quartile 2 | 16/177 | 0.61 (0.25, 1.49) | 16/159 | 2.26 (0.78, 6.57) |  |
| Quartile 3 | 10/181 | 0.54 (0.20, 1.47) | 7/172 | 0.79 (0.21, 2.93) |  |
| Quartile 4 | 5/169 | 0.25 (0.07, 0.88) | 8/188 | 0.30 (0.08, 1.17) |  |
| *P* for trend |  | 0.032 |  | 0.049 |  |
| Lycopene |  |  |  |  | 0.454 |
| Per 1-SD increase |  | 1.10 (0.78, 1.54) |  | 0.87 (0.53, 1.44) |  |
| Quartile 1 | 12/187 | 1.00 (Ref) | 17/153 | 1.00 (Ref) |  |
| Quartile 2 | 13/170 | 0.92 (0.33, 2.55) | 9/177 | 0.72 (0.25, 2.10) |  |
| Quartile 3 | 16/180 | 1.66 (0.63, 4.39) | 9/165 | 0.75 (0.24, 2.31) |  |
| Quartile 4 | 10/187 | 1.28 (0.45, 3.64) | 8/165 | 0.85 (0.27, 2.69) |  |
| *P* for trend |  | 0.422 |  | 0.758 |  |
| Lutein/zeaxanthin |  |  |  |  | 0.023 |
| Per 1-SD increase |  | 1.27 (0.96, 1.69) |  | 0.64 (0.38, 1.10) |  |
| Quartile 1 | 13/181 | 1.00 (Ref) | 19/157 | 1.00 (Ref) |  |
| Quartile 2 | 14/191 | 0.77 (0.28, 2.13) | 7/156 | 0.51 (0.16, 1.58) |  |
| Quartile 3 | 11/182 | 0.82 (0.29, 2.35) | 9/165 | 0.61 (0.18, 2.02) |  |
| Quartile 4 | 13/170 | 1.06 (0.37, 2.98) | 8/182 | 0.35 (0.11, 1.18) |  |
| *P* for trend |  | 0.868 |  | 0.118 |  |
| Total carotenoid |  |  |  |  | 0.080 |
| Per 1-SD increase |  | 0.97 (0.61, 1.54) |  | 0.48 (0.25, 0.92) |  |
| Quartile 1 | 17/202 | 1.00 (Ref) | 16/135 | 1.00 (Ref) |  |
| Quartile 2 | 16/177 | 0.91 (0.35, 2.34) | 13/162 | 1.20 (0.42, 3.43) |  |
| Quartile 3 | 11/187 | 1.37 (0.52, 3.62) | 8/164 | 0.81 (0.26, 2.56) |  |
| Quartile 4 | 7/158 | 0.87 (0.28, 2.70) | 6/199 | 0.34 (0.09, 1.22) |  |
| *P* for trend |  | 0.922 |  | 0.097 |  |

*P* for trend were obtained by including quartile number as a continuous variable in the regression model. *P* for interaction was calculated using the likelihood ratio test. Models were adjusted for age (continuous), race/ethnicity (non-Hispanic white, black, Mexican-American, other Hispanic, or other race/ethnicity), education level (less than high school, high school or equivalent, college or above, or missing), family income-to-poverty ratio (<1.3, 1.3 to ≤3.5, >3.5, or missing), body mass index (<18.5, 18.5 to <25, 25 to <30, ≥30 kg/m^2^, or missing), drinking status (nondrinker or drinker), smoking status (never smoker, former smoker, current smoker, or missing), HbA_1c_ (<6.5%, ≥6.5%, or missing), hypertension (yes, no, or missing), hypercholesterolemia (yes, no, or missing), and cancer (yes, no, or missing).

**Supplementary Table 9.** Association between serum carotenoids concentrations and risk of age-related macular degeneration among middle-aged and older adults by sex

| Quartiles of serum carotenoids concentrations | Male | | Female | | *P* for interaction |
| --- | --- | --- | --- | --- | --- |
|  | Cases/controls | OR (95% CI) | Cases/controls | OR (95% CI) |  |
| α-carotene |  |  |  |  | 0.527 |
| Per 1-SD increase |  | 1.18 (0.88, 1.57) |  | 1.02 (0.72, 1.43) |  |
| Quartile 1 | 15/200 | 1.00 (Ref) | 10/120 | 1.00 (Ref) |  |
| Quartile 2 | 18/193 | 0.85 (0.39, 1.84) | 9/163 | 0.42 (0.14, 1.23) |  |
| Quartile 3 | 18/172 | 0.78 (0.35, 1.73) | 12/170 | 0.56 (0.20, 1.56) |  |
| Quartile 4 | 14/145 | 0.72 (0.30, 1.70) | 14/205 | 0.68 (0.24, 1.89) |  |
| *P* for trend |  | 0.439 |  | 0.693 |  |
| β-carotene |  |  |  |  | 0.955 |
| Per 1-SD increase |  | 1.23 (0.94, 1.62) |  | 1.22 (0.99, 1.51) |  |
| Quartile 1 | 15/226 | 1.00 (Ref) | 5/118 | 1.00 (Ref) |  |
| Quartile 2 | 24/179 | 1.60 (0.77, 3.31) | 10/162 | 1.00 (0.30, 3.32) |  |
| Quartile 3 | 11/182 | 0.52 (0.22, 1.24) | 7/169 | 0.47 (0.13, 1.76) |  |
| Quartile 4 | 15/123 | 1.06 (0.46, 2.46) | 23/209 | 1.49 (0.47, 4.73) |  |
| *P* for trend |  | 0.410 |  | 0.385 |  |
| β-cryptoxanthin |  |  |  |  | 0.963 |
| Per 1-SD increase |  | 0.85 (0.58, 1.26) |  | 0.84 (0.51, 1.38) |  |
| Quartile 1 | 14/203 | 1.00 (Ref) | 14/139 | 1.00 (Ref) |  |
| Quartile 2 | 16/177 | 1.29 (0.59, 2.82) | 6/169 | 0.39 (0.13, 1.14) |  |
| Quartile 3 | 23/168 | 1.56 (0.73, 3.32) | 16/163 | 1.23 (0.50, 3.04) |  |
| Quartile 4 | 12/162 | 0.90 (0.37, 2.16) | 9/187 | 0.53 (0.19, 1.46) |  |
| *P* for trend |  | 0.973 |  | 0.567 |  |
| Lycopene |  |  |  |  | 0.096 |
| Per 1-SD increase |  | 0.86 (0.63, 1.17) |  | 0.53 (0.33, 0.86) |  |
| Quartile 1 | 18/181 | 1.00 (Ref) | 17/153 | 1.00 (Ref) |  |
| Quartile 2 | 19/164 | 1.22 (0.59, 2.54) | 16/170 | 0.96 (0.43, 2.14) |  |
| Quartile 3 | 19/177 | 1.23 (0.58, 2.63) | 10/164 | 0.67 (0.27, 1.68) |  |
| Quartile 4 | 9/188 | 0.54 (0.22, 1.32) | 2/171 | 0.14 (0.03, 0.67) |  |
| *P* for trend |  | 0.266 |  | 0.013 |  |
| Lutein/zeaxanthin |  |  |  |  | 0.173 |
| Per 1-SD increase |  | 1.42 (1.15, 1.75) |  | 1.05 (0.71, 1.55) |  |
| Quartile 1 | 15/179 | 1.00 (Ref) | 10/166 | 1.00 (Ref) |  |
| Quartile 2 | 18/187 | 1.07 (0.49, 2.33) | 14/149 | 1.47 (0.54, 4.00) |  |
| Quartile 3 | 16/177 | 1.01 (0.46, 2.25) | 12/162 | 1.28 (0.46, 3.55) |  |
| Quartile 4 | 16/167 | 1.06 (0.48, 2.37) | 9/181 | 1.12 (0.37, 3.35) |  |
| *P* for trend |  | 0.927 |  | 0.966 |  |
| Total carotenoid |  |  |  |  | 0.757 |
| Per 1-SD increase |  | 1.18 (0.88, 1.58) |  | 1.10 (0.80, 1.50) |  |
| Quartile 1 | 19/200 | 1.00 (Ref) | 13/138 | 1.00 (Ref) |  |
| Quartile 2 | 17/176 | 0.97 (0.46, 2.05) | 8/167 | 0.48 (0.17, 1.33) |  |
| Quartile 3 | 14/184 | 0.69 (0.31, 1.51) | 11/161 | 0.57 (0.21, 1.52) |  |
| Quartile 4 | 15/150 | 0.92 (0.41, 2.04) | 13/192 | 0.80 (0.30, 2.16) |  |
| *P* for trend |  | 0.620 |  | 0.801 |  |

*P* for trend were obtained by including quartile number as a continuous variable in the regression model. *P* for interaction was calculated using the likelihood ratio test. Models were adjusted for age (continuous), race/ethnicity (non-Hispanic white, black, Mexican-American, other Hispanic, or other race/ethnicity), education level (less than high school, high school or equivalent, college or above, or missing), family income-to-poverty ratio (<1.3, 1.3 to ≤3.5, >3.5, or missing), body mass index (<18.5, 18.5 to <25, 25 to <30, ≥30 kg/m^2^, or missing), drinking status (nondrinker or drinker), smoking status (never smoker, former smoker, current smoker, or missing), HbA_1c_ (<6.5%, ≥6.5%, or missing), hypertension (yes, no, or missing), hypercholesterolemia (yes, no, or missing), and cancer (yes, no, or missing).

**Supplementary Table 10.** Association between serum carotenoids concentrations and risk of any ocular disease among middle-aged and older adults by smoking status

| Quartiles of serum carotenoids concentrations | Never | | Former or current smoker | | *P* for interaction |
| --- | --- | --- | --- | --- | --- |
|  | Cases/controls | OR (95% CI) | Cases/controls | OR (95% CI) |  |
| **α-carotene** |  |  |  |  | 0.026 |
| Per 1-SD increase |  | 1.03 (0.82, 1.30) |  | 0.69 (0.53, 0.89) |  |
| Quartile 1 | 39/71 | 1.00 (Ref) | 73/161 | 1.00 (Ref) |  |
| Quartile 2 | 53/108 | 0.70 (0.38, 1.29) | 73/148 | 1.17 (0.74, 1.84) |  |
| Quartile 3 | 51/139 | 0.51 (0.27, 0.96) | 64/116 | 0.99 (0.61, 1.62) |  |
| Quartile 4 | 47/152 | 0.52 (0.27, 1.00) | 36/142 | 0.44 (0.25, 0.76) |  |
| *P* for trend |  | 0.0441 |  | 0.007 |  |
| **β-carotene** |  |  |  |  | 0.346 |
| Per 1-SD increase |  | 1.01 (0.85, 1.22) |  | 0.89 (0.71, 1.10) |  |
| Quartile 1 | 36/81 | 1.00 (Ref) | 80/166 | 1.00 (Ref) |  |
| Quartile 2 | 45/105 | 0.81 (0.43, 1.51) | 70/152 | 0.88 (0.56, 1.37) |  |
| Quartile 3 | 57/145 | 0.61 (0.34, 1.13) | 43/124 | 0.54 (0.32, 0.90) |  |
| Quartile 4 | 52/139 | 0.62 (0.32, 1.18) | 53/125 | 0.55 (0.33, 0.93) |  |
| P for trend |  | 0.104 |  | 0.008 |  |
| **β-cryptoxanthin** |  |  |  |  | 0.151 |
| Per 1-SD increase |  | 0.81 (0.64, 1.03) |  | 1.02 (0.84, 1.23) |  |
| Quartile 1 | 44/80 | 1.00 (Ref) | 74/171 | 1.00 (Ref) |  |
| Quartile 2 | 51/108 | 1.24 (0.69, 2.23) | 60/147 | 0.80 (0.49, 1.29) |  |
| Quartile 3 | 48/119 | 1.03 (0.56, 1.89) | 67/136 | 1.12 (0.70, 1.79) |  |
| Quartile 4 | 47/163 | 0.71 (0.38, 1.32) | 45/113 | 0.72 (0.42, 1.24) |  |
| *P* for trend |  | 0.178 |  | 0.554 |  |
| **Lycopene** |  |  |  |  | 0.615 |
| Per 1-SD increase |  | 0.96 (0.77, 1.19) |  | 0.89 (0.73, 1.08) |  |
| Quartile 1 | 59/90 | 1.00 (Ref) | 84/133 | 1.00 (Ref) |  |
| Quartile 2 | 53/110 | 0.98 (0.57, 1.69) | 60/145 | 0.68 (0.43, 1.08) |  |
| Quartile 3 | 46/112 | 1.12 (0.63, 1.99) | 64/147 | 0.96 (0.61, 1.52) |  |
| Quartile 4 | 32/158 | 0.74 (0.40, 1.35) | 38/142 | 0.61 (0.36, 1.02) |  |
| *P* for trend |  | 0.435 |  | 0.177 |  |
| **Lutein/zeaxanthin** |  |  |  |  | 0.889 |
| Per 1-SD increase |  | 1.03 (0.84, 1.26) |  | 1.01 (0.85, 1.19) |  |
| Quartile 1 | 52/102 | 1.00 (Ref) | 63/152 | 1.00 (Ref) |  |
| Quartile 2 | 57/97 | 1.22 (0.69, 2.15) | 58/155 | 0.88 (0.54, 1.44) |  |
| Quartile 3 | 43/115 | 0.68 (0.37, 1.25) | 74/133 | 1.48 (0.91, 2.39) |  |
| Quartile 4 | 38/156 | 0.56 (0.31, 1.02) | 51/127 | 0.84 (0.50, 1.39) |  |
| *P* for trend |  | 0.015 |  | 0.928 |  |
| **Total carotenoid** |  |  |  |  | 0.440 |
| Per 1-SD increase |  | 0.97 (0.79, 1.20) |  | 0.87 (0.71, 1.06) |  |
| Quartile 1 | 44/81 | 1.00 (Ref) | 87/157 | 1.00 (Ref) |  |
| Quartile 2 | 62/103 | 1.78 (0.99, 3.19) | 60/140 | 0.77 (0.48, 1.23) |  |
| Quartile 3 | 43/128 | 0.91 (0.49, 1.68) | 54/145 | 0.66 (0.41, 1.06) |  |
| Quartile 4 | 41/158 | 0.79 (0.42, 1.48) | 45/125 | 0.62 (0.37, 1.04) |  |
| *P* for trend |  | 0.104 |  | 0.045 |  |

*P* for trend were obtained by including quartile number as a continuous variable in the regression model. *P* for interaction was calculated using the likelihood ratio test. Models were adjusted for age (continuous), sex (male or female), race/ethnicity (non-Hispanic white, black, Mexican-American, other Hispanic, or other race/ethnicity), education level (less than high school, high school or equivalent, college or above, or missing), family income-to-poverty ratio (<1.3, 1.3 to ≤3.5, >3.5, or missing), body mass index (<18.5, 18.5 to <25, 25 to <30, ≥30 kg/m^2^, or missing), drinking status (nondrinker or drinker), HbA1c (<6.5%, ≥6.5%, or missing), hypertension (yes, no, or missing), hypercholesterolemia (yes, no, or missing), and cancer (yes, no, or missing).
